# Heterogeneity of soil bacterial and bacteriophage communities in three rice agroecosystems and potential impacts of bacteriophage on nutrient cycling

**DOI:** 10.1186/s40793-022-00410-8

**Published:** 2022-04-06

**Authors:** Yajiao Wang, Yu Liu, Yuxing Wu, Nan Wu, Wenwen Liu, Xifeng Wang

**Affiliations:** 1 State Key Laboratory for Biology of Plant Diseases and Insect Pests, Institute of Plant Protection, Chinese Academy of Agricultural Sciences, Beijing, 100193 China; 2 Institute of Plant Protection, Hebei Academy of Agricultural and Forestry Sciences, Baoding, 071000 China

**Keywords:** Paddy soil, Bacteriophage, Bacteriophage–bacteria interaction, Auxiliary metabolic genes, Nitrogen cycling

## Abstract

**Background:**

As genetic entities infecting and replicating only in bacteria, bacteriophages can regulate the community structure and functions of their host bacteria. The ecological roles of bacteriophages in aquatic and forest environments have been widely explored, but those in agroecosystems remains limited. Here, we used metagenomic sequencing to analyze the diversity and interactions of bacteriophages and their host bacteria in soils from three typical rice agroecosystems in China: double cropping in Guangzhou, southern China, rice–wheat rotation cropping in Nanjing, eastern China and early maturing single cropping in Jiamusi, northeastern China. *Enterobacter* phage-NJ was isolated and its functions on soil nitrogen cycling and effect on soil bacterial community structure were verified in pot inoculation experiments and 16S rRNA gene sequencing.

**Results:**

Soil bacterial and viral diversity and predicted functions varied among the three agroecosystems. Genes detected in communities from the three agroecosystems were associated with typical functions: soil bacteria in Jiamusi were significantly enriched in genes related to carbohydrate metabolism, in Nanjing with xenobiotics biodegradation and metabolism, and in Guangzhou with virulence factors and scarce in secondary metabolite biosynthesis, which might lead to a significant occurrence of rice bacterial diseases. The virus community structure varies significantly among the three ecosystems, only 13.39% of the total viral species were shared by the three rice agroecosystems, 59.56% of the viral species were specific to one agroecosystem. Notably, over-represented auxiliary carbohydrate-active enzyme (CAZyme) genes were identified in the viruses, which might assist host bacteria in metabolizing carbon, and 67.43% of these genes were present in Jiamusi. In bacteriophage isolation and inoculation experiments, *Enterobacter* bacteriophage-NJ reduced the nitrogen fixation capacity of soil by lysing N-fixing host bacteria and changed the soil bacterial diversity and community structure.

**Conclusion:**

Our results showed that diversity and function predicted of paddy soil bacteria and viruses varied in the three agroecosystems. Soil bacteriophages can affect nutrient cycling by boosting host metabolism through the carried auxiliary metabolic genes (AMGs) and lysing the host bacteria that are involved in biogeochemical cycles. These findings form a basis for better understanding bacterial and bacteriophage diversity in different rice agroecosystems, laying a solid foundation for further studies of soil microbial communities that support ecofriendly production of healthy rice.

**Supplementary Information:**

The online version contains supplementary material available at 10.1186/s40793-022-00410-8.

## Background

Rice, the primary staple food for about 60% of China’s population [1, 2] and planted in about 29.5 million ha in China, accounts for 35% of the total area planted in grain crops [3]. Rice-growing areas in China are quite diverse and distributed throughout most of the country and differ in ecological environment, cultivation methods and production levels. The three most important rice agroecosystems are rice double cropping in southern China, which has a subtropical and marginal tropical monsoon climate, rice rotation with other crops in central and eastern China, which has a hot and rainy summer but cold and less raining winter and early-maturing single cropping in northeastern China, which has a cold, long winter and warm, short summer. Different ecological environments and farming methods lead to different soil physicochemical properties and soil microbial diversity [4–6].

The hundreds of millions of soil microbes in soils include bacteria, fungi, archaea, viruses, that compete for nutrients and living space in the soil. Determining the physicochemical properties of soils and their effects on the soil microbial composition and diversity in different regions can help in utilizing local natural resources effectively and improving cultivation techniques to safely produce higher-yielding rice. Soil microbes not only breakdown organic matter and minerals, they also secrete secondary metabolites that can regulate plant growth [7–9]. Microbes in soil can cause disease or help protect against disease [10, 11]. Soil microbial communities and functions have strong spatial variability [12]. Bahram et al*.* (2018) found contrasting patterns in bacterial diversity in top soils across a latitudinal gradient around the world, with the highest diversity in temperate habitats, and environmental variables caused greater variation in microbial gene diversity than geographic distance did [13]. Soil microbial diversity and multifunctionality (decomposition and nutrient cycling, plant productivity) were demonstrated to be spatially specific and positively correlated in 78 global drylands and 179 locations across Scotland [14]. Elucidating spatial and temporal patterns of soil microbial diversity, community composition, and function will thus contribute to a better understanding of the generation and maintenance of microbial diversity, which will help to harness beneficial microbial resources for full use of their ecological functions in promoting healthy growth of plants in environmentally safe ecosystems.

Bacteriophages, which can infect and replicate within particular bacterial hosts, are important components of soil microbial community [15]. Bacteriophages can regulate the community structure and function of bacteria and even the whole soil microbes [16], modulate host bacterial populations and diversities by lysing bacterial cells [17], drive host evolution via bacteriophage-mediated horizontal gene transfer [18], and alter host bacterial function by reprogramming their metabolism via the expression of virus-encoded auxiliary metabolic genes (AMGs) [19]. AMGs have a variety of metabolic functions, including carbon, nitrogen and sulfur metabolism [20, 21], and photosynthesis [22]. However, most studies on the function of bacteriophages have focused on marine ecosystems or the human intestinal tract rather than soils in agroecosystems.

Our knowledge about paddy soil microbial diversity and function in different agroecosystems is limited, and even less is known about viral communities and their roles. Studies of viruses in glacier soil [23], mangrove soil [24], mud volcanic soil [25], and Antarctic soil [26] revealed differences in soil viral community structure and largely uncharacterized viral assemblages. In the present study, we used metagenomic sequencing to analyze bacterial and viral community composition, diversity and ecological functions in paddy bulk soils in the three most important rice agroecosystems, double cropping in southern China, rice–wheat rotation cropping in central China, and early-maturing single cropping in northeastern China. We also tested the effects of bacteriophages on soil bacterial community structure and function in amended soils in pot experiments.

## Results

### Soil microbial communities and differences in three rice agroecosystems

**Fig. 1:**
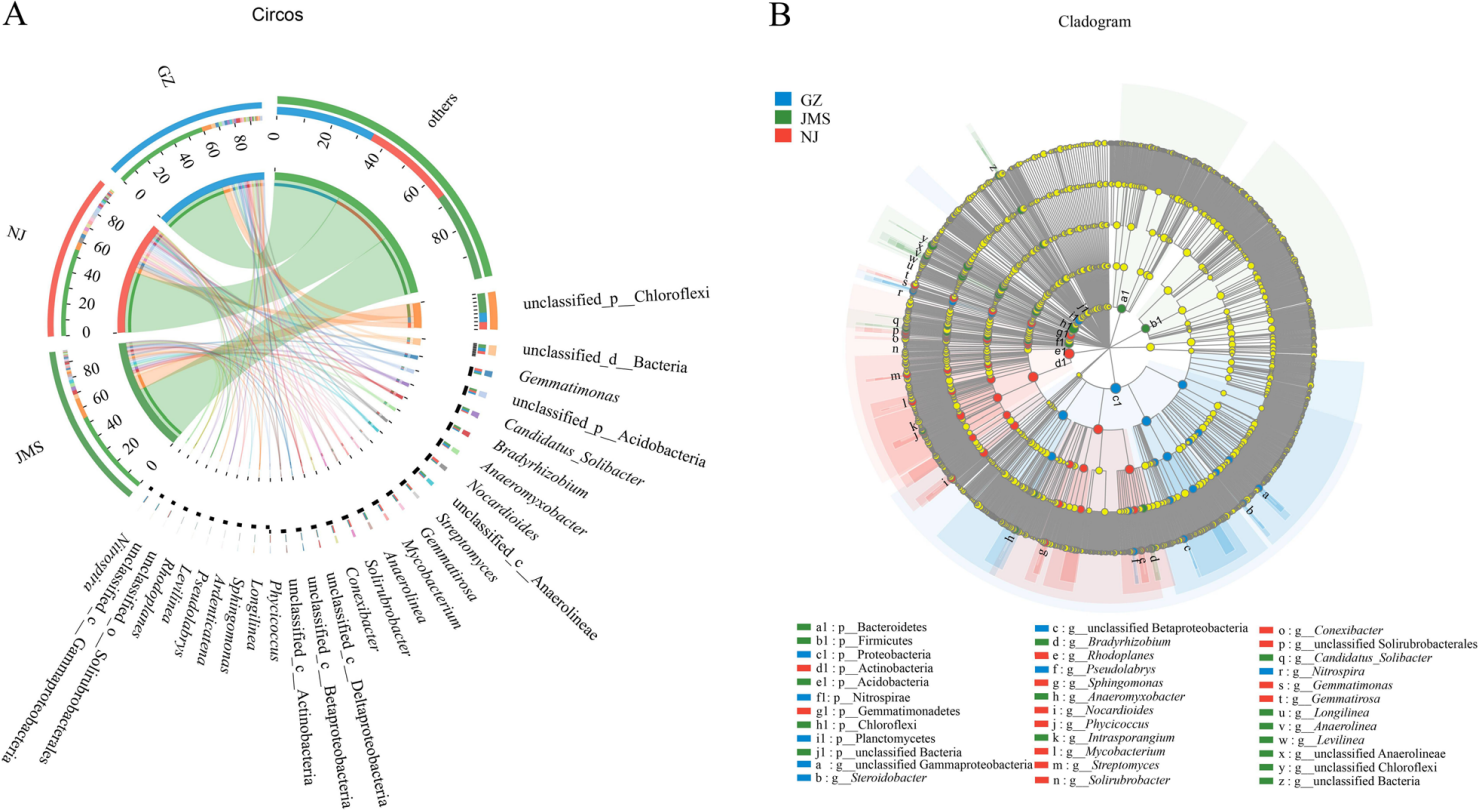
Differential analysis of soil microbial composition in three rice agroecosystems in China. **A** Circos analysis of distribution and proportion of the microbial dominant genera. In top left half of circle, numbers are relative abundance of genera in the three agroecosystems (GZ: rice double cropping in Guangzhou; JMS: rice single cropping in Jiamusi; NJ: rice–wheat rotation cropping in Nanjing; others: genera with abundance less than 1% were merged and indicated as others). In lower right, the axis represents the distribution proportion of a genus in each agroecosystem. **B** Linear discriminant effect size analysis identified remarkable enrichment of taxa from phylum to genus level. Different color nodes represent the microbial groups that are significantly higher in the corresponding groups and have a significant influence on the difference between the groups. Light yellow nodes represent the microbial groups that contribute no significant difference in different groups or influence on the difference between groups

In the metagenomic sequencing, 1.10 billion raw reads were obtained. After trimming, 1.07 billion clean reads remained. In Guangzhou, Nanjing and Jiamusi, 511,481, 593,018, 848,794 contigs were obtained, respectively, after clean reads were assembled. Based on similarities to entries in the NCBI-NR database, 97.81% of sequences were classified as bacteria, 1.73% as archaea, 0.27% as eukaryota, and 0.026% as viruses. In total, 82 phyla, 1,904 genera and 12,455 microbial species were identified. At the phylum level, Proteobacteria, Actinobacteria, Chloroflexi, Acidobacteria and Bacteroidetes were the top five most-abundant phyla, accounting for more than 78% of all the sequences (Additional file 1: Fig. S1A). According to the LEfSe analysis (LDA > 3.5, *p* < 0.05), soils in Guangzhou and Nanjing were enriched in microbes belonging to Proteobacteria and Actinobacteria, respectively, while soils in Jiamusi were enriched in bacteria belonging to Chloroflexi, Acidobacteria and Bacteroidetes (Fig. 1A). At the generic level, there were significant differences in the dominant genera (with abundance greater than 1% and without unclassified genera). There were 10 dominant genera in Guangzhou, 13 in Nanjing and 9 in Jiamusi, only 20% of the total dominant genera were shared by the three rice agroecosystems, and 45% were unique to a single rice agroecosystem (Fig. 1B, Additional file 1: Table S3). The top three most-dominant genera were *Gemmatimonas*, *Anaeromyxobacter* and *Candidatus Solibacter* in Guangzhou, *Nocardioides*, *Gemmatimonas* and *Solirubrobacter* in Nanjing, *Bradyrhizobium*, *Candidatus Solibacter* and *Anaeromyxobacter* in Jiamusi (Additional file 1: Fig. S1B). It is worth noting that none was shared by the three rice agroecosystems. Our results that the microbial community composition differed among the three rice agroecosystems was confirmed by PCoA analysis. The principal coordinate axis 1 (PCo1) and the principal coordinate axis 2 (PCo2) explained, respectively, 26.66% and 19.22% of the variation in microbial species (Additional file 1: Fig. S2).

At the species level, we analyzed the relative abundance of four main pathogenic bacteria: *Pseudomonas syringae*, *Acidovorax avenae*, *Xanthomonas oryzae* and *Dickeya zeae*, the results showed that the relative abundance of the four pathogens was the highest in Guangzhou (Additional file 1: Fig. S3A). Similarly, in the field disease evaluation, we found that the disease indices for rice brown spot, brown streak, bacterial blight, bacterial leaf streak and foot rot were the highest in Guangzhou (Additional file 1: Fig. S3B).

### Complicated relationship between bacteriophage and bacterial abundance and diversity

**Fig. 2:**
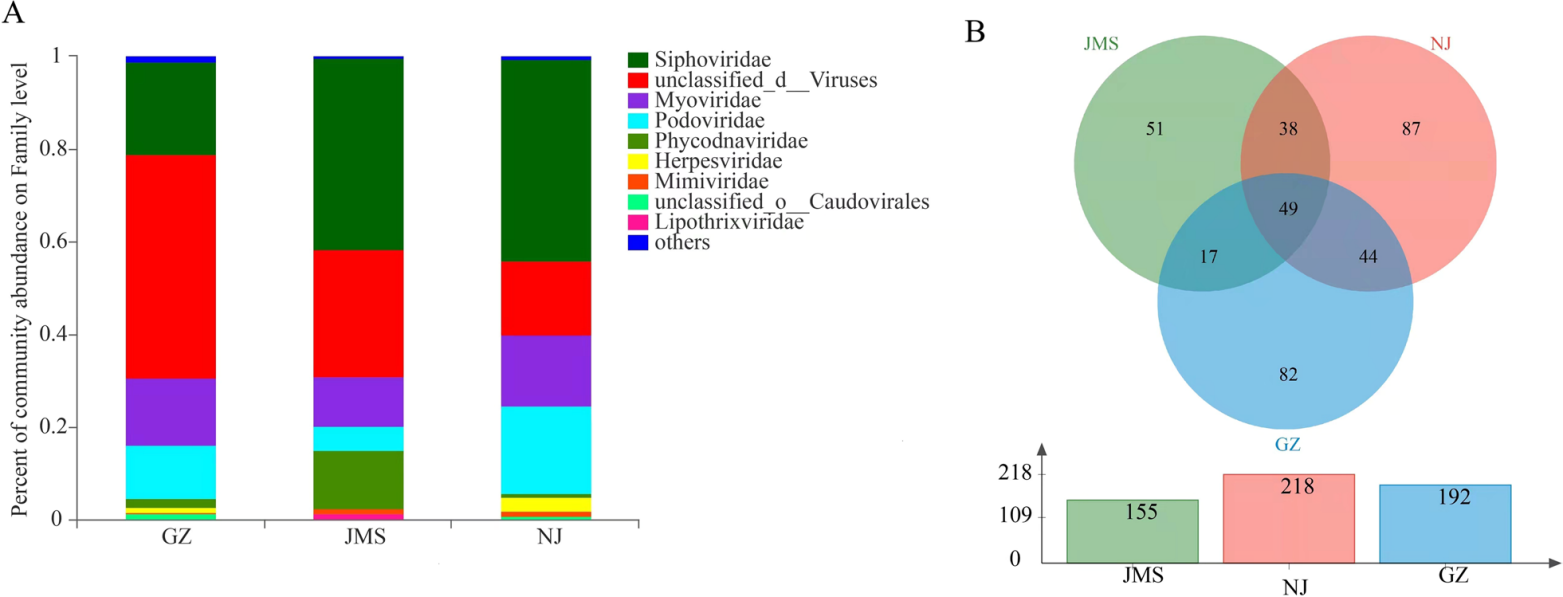
Differential analysis of soil viral composition in three rice agroecosystems in China. **A** Relative abundance of main soil viral families in the three rice agroecosystems in China. **B** Venn diagram showing the number of viral species shared and unique among different rice agroecosystems. GZ: rice double cropping in Guangzhou; JMS: rice single cropping in Jiamusi; NJ: rice–wheat rotation cropping in Nanjing

Viral and bacterial genes were selected out to analysis their relationship. Metagenomic sequencing only detected viruses that were relatively abundant. In total, 14 families, 29 genera and 368 species of viruses were identified. As in the most-often characterized viromes in various habitats, the annotated viruses in three rice agroecosystems belonged to families Siphoviridae, Podoviridae, Myoviridae, Phycodnaviridae and Herpesviridae. The relative abundance of Phycodnaviridae was highest in Jiamusi, and Siphoviridae, Podoviridae, Myoviridae and Herpesviridae were highest in Nanjing (Fig. 2A). Similar with the trend for the number of bacterial species, the number of virus species was highest in Nanjing (218 virus species, 11,693 bacterial species), followed by Guangzhou (192 virus species, 11,506 bacterial species) and Jiamusi (155 virus species, 11,424 bacterial species). The three rice agroecosystems shared 13.39% of the total virus species, and 27.05% of the total virus species were shared by two agroecosystems; however, 59.56% of the virus species were specific to one agroecosystem.

Bacteriophages that infect the same bacterial genus were grouped together. We analyzed the correlation between the relative abundance of dominant bacteriophage groups (relative abundance greater than 1%) and their host bacterial genera (Table 1). There were 19 dominant bacteriophage groups, the relative abundance of the hosts for three bacteriophage groups was greater than 1%, less than 0.01% for four bacteriophage groups, and between 0.01 and 1% for 12 bacteriophage groups. Thus, the dominant bacteriophages were mainly distributed in bacteria with moderate abundance. The relationship between the relative abundance of the bacteriophages and that of their hosts is complex. The ratio of viral relative abundance to bacterial relative abundance (VBR) varies widely, between 0 and 27,382. But when VBR was greater than 100 in at least one of the three cropping systems, the abundance of bacteriophage and host bacteria was negatively correlated (*ρ* < − 0.5).

**Table 1:** Correlation between the relative abundance of dominant bacteriophage groups and the host bacterial genera in the three rice agroecosystems studied in China

Phages	NJ phage	NJ bacteria	NJ VBR	GZ phage	GZ bacteria	GZ VBR	JMS phage	JMS bacteria	JMS VBR	*ρ*	*p*
*Achromobacter* phage	1.247 ± 0.512	0.845 ± 0.035	1.476	0.252 ± 0.057	1.370 ± 0.039	0.184	0	1.854 ± 0.073	0	− 0.855	0.001
*Vibrio* phage	4.007 ± 1.022	0.024 ± 0.001	164.991	3.067 ± 0.668	0.031 ± 0.0003	98.523	0.254 ± 0.149	0.036 ± 0.001	7.1	− 0.856	0.001
*Gordonia* phage	3.759 ± 1.168	0.080 ± 0.003	47.181	0.507 ± 0.577	0.058 ± 0.002	8.705	20.029 ± 2.19	0.043 ± 0.001	469.223	− 0.708	0.01
*Streptomyces* phage	0.865 ± 0.365	1.977 ± 0.056	0.438	2.955 ± 0.823	1.127 ± 0.059	2.622	2.915 ± 1.688	1.136 ± 0.066	2.566	− 0.704	0.011
*Enterobacter* phage	15.889 ± 0.484	0.005 ± 0.0006	3050.137	0.128 ± 0.101	0.008 ± 0.0007	15.398	0	0.007 ± 0.001	0	− 0.684	0.014
*Stenotrophomonas* phage	7.319 ± 1.331	0.040 ± 0.001	184.654	3.905 ± 1.262	0.045 ± 0.001	87.197	0	0.044 ± 0.0005	0	− 0.591	0.043
*Salmonella* phage	5.390 ± 0.478	0.002 ± 0.0003	2515.275	0.739 ± 0.326	0.003 ± 0.0005	252.934	0	0.003 ± 0.0003	0	− 0.572	0.049
*Paramecium bursaria* phage	0.401 ± 0.276	0.0009 ± 0.0002	469.215	1.542 ± 0.206	0.0003 ± 0.000007	5020.489	10.980 ± 1.297	0.0004 ± 0.000005	27,382.718	− 0.509	0.091
*Rhodococcus* phage	1.827 ± 1.079	0.393 ± 0.012	4.652	3.983 ± 2.79	0.196 ± 0.009	20.372	3.930 ± 1.682	0.181 ± 0.012	21.693	− 0.357	0.254
*Burkholderia* phage	7.139 ± 1.112	0.226 ± 0.003	31.647	3.873 ± 0.770	0.347 ± 0.005	11.153	0.232 ± 0.186	0.232 ± 0.002	1.002	− 0.018	0.955
*Caulobacter* phage	0.374 ± 0.278	0.087 ± 0.004	4.293	0.972 ± 0.531	0.099 ± 0.002	9.751	0.701 ± 0.201	0.058 ± 0.002	12.128	0.204	0.525
*Mycobacterium* phage	8.404 ± 7.98	2.405 ± 0.133	3.495	5.269 ± 0.545	1.278 ± 0.087	4.122	5.679 ± 1.066	0.438 ± 0.027	12.961	0.263	0.409
*Bacillus* phage	2.181 ± 0.428	0.214 ± 0.002	10.170	1.106 ± 0.518	0.240 ± 0.002	4.609	2.670 ± 1.117	0.282 ± 0.005	9.452	0.332	0.291
*Synechococcus* phage	1.318 ± 0.377	0.059 ± 0.0005	22.252	2.990 ± 0.831	0.082 ± 0.001	36.361	3.436 ± 1.065	0.063 ± 0.001	54.548	0.343	0.275
*Pseudomonas* phage	6.964 ± 2.194	0.247 ± 0.013	28.207	5.904 ± 1.422	0.334 ± 0.007	17.651	2.486 ± 1.049	0.207 ± 0.002	11.984	0.477	0.116
*Ralstonia* phage	0.065 ± 0.089	0.063 ± 0.002	1.029	1.046 ± 0.516	0.117 ± 0.0008	8.933	0.501 ± 0.102	0.076 ± 0.001	6.636	0.563	0.046
*Rhodoferax* phage	0	0.027 ± 0.001	0	1.392 ± 0.386	0.054 ± 0.004	25.709	0	0.044 ± 0.0007	0	0.778	0.003
*Actinoplanes* phage	1.025 ± 0.482	0.298 ± 0.007	3.446	0.045 ± 0.089	0.147 ± 0.009	0.303	0.523 ± 0.295	0.173 ± 0.011	3.031	0.781	0.003
*Arthrobacter* phage	4.391 ± 0.892	0.400 ± 0.015	10.969	2.286 ± 0.579	0.118 ± 0.004	19.392	16.613 ± 1.227	0.547 ± 0.079	30.394	0.837	0.001

GZ: rice double cropping system in Guangzhou; JMS: rice single cropping system in Jiamusi; NJ: rice–wheat rotation cropping ecosystem in Nanjing. VBR: the ratio of viral relative abundance to bacterial relative abundance

**Fig. 3:**
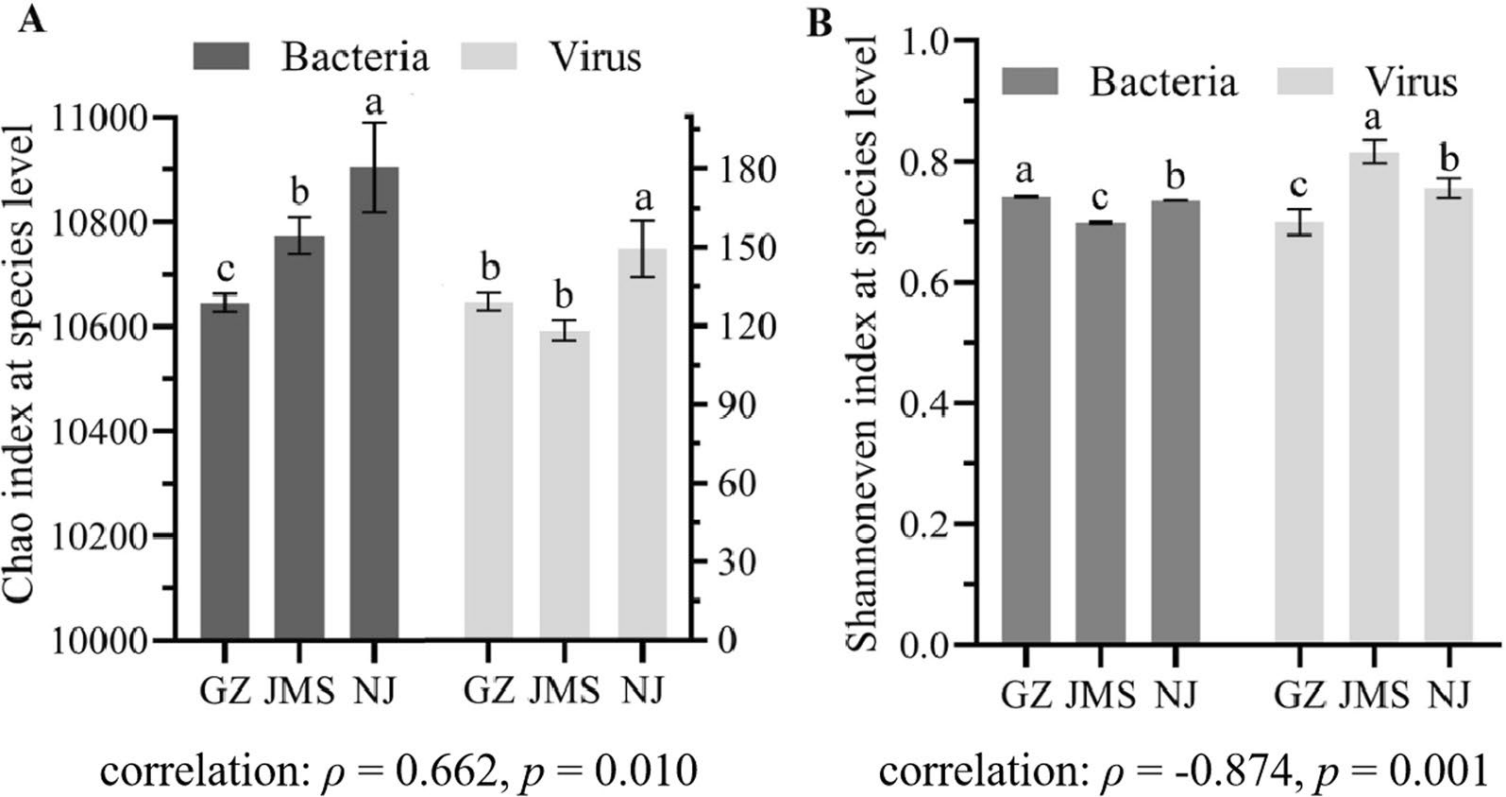
Alpha diversity of soil bacterial and viral communities. **A** Chao index of soil bacterial community and viral community. **B** Shannon index of soil bacterial community and viral community. GZ: rice double cropping in Guangzhou; JMS: rice single cropping in Jiamusi; NJ: rice–wheat rotation cropping in Nanjing

Metagenomic analysis of richness (represented by the Chao index) and diversity (represented by the Shannon index) revealed a significant difference in the bacterial and bacteriophage communities of paddy soils in different cropping systems. The Chao index showed that the bacterial richness in Nanjing was significantly higher than in Jiamusi and Guangzhou, while the difference between Jiamusi and Guangzhou was not significant. The viral richness was positively correlated with that of bacteria (*ρ* = 0.662, *p* = 0.019) (Fig. 3A), and the viral richness of Nanjing was significantly higher than that of Jiamusi and Guangzhou. The Shannon index analysis found that the bacterial diversity of Nanjing and Guangzhou did not differ significantly, but both were significantly higher than in Jiamusi. Viral diversity was negatively correlated with that of bacteria (*ρ* = − 0.876, *p* = 0.001) (Fig. 3B).

### Soil bacterial function and its relationship with environment in three agroecosystems

**Fig. 4:**
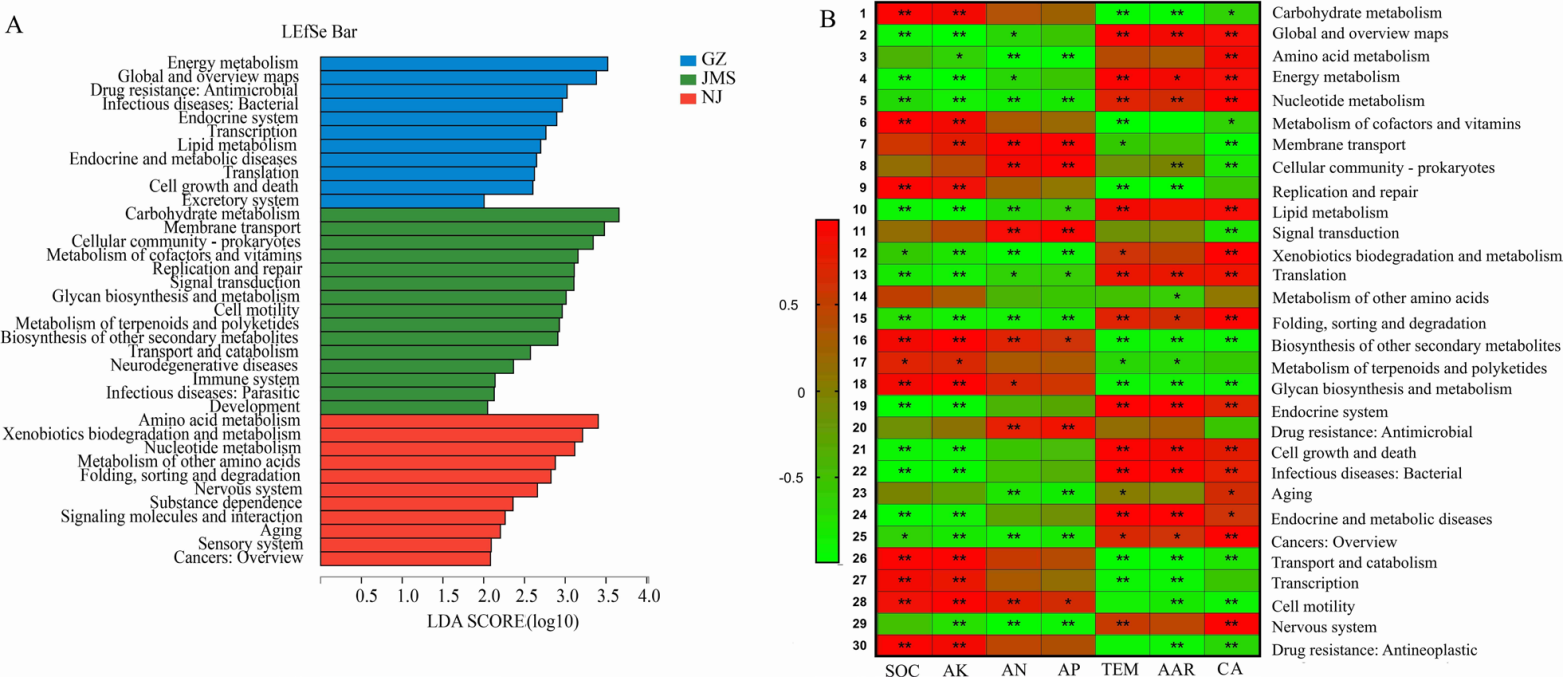
Analysis of differences in soil bacterial functions and correlation analysis with environmental factors in the three rice agroecosystems. **A** Linear discriminant analysis of soil bacterial function at KEGG level 2. **B** Redundancy analysis showing the influence of environmental factors on soil bacterial function at KEGG level 2. GZ: rice double cropping in Guangzhou; JMS: rice single cropping in Jiamusi; NJ: rice–wheat rotation cropping in Nanjing. SOC: soil organic carbon; AK: available potassium, AN: available nitrogen; AP: available phosphorus; TEM: annual accumulated temperature; AAR: annual precipitation; CA: chemical pesticides application

In the functional annotation of the soil metagenomic reads for the bacterial community using the Kyoto Encyclopedia of Genes and Genomes (KEGG) database, most sequences were associated with metabolism (70.24–72.38%), and far fewer were involved in genetic information processing (8.44–8.93%), environmental information processing (6.79–7.26%), cellular processe**s** (5.49–6.01%), human diseases (3.93–4.57%), and organismal systems (2.71%-2.85%). According to the LEfSe analysis (Fig. 4A), soil bacteria in Jiamusi were significantly enriched in genes associated with carbohydrate metabolism, replication and repair, biosynthesis of other secondary metabolites which were positively correlated with a high soil organic carbon (SOC) content (Fig. 4B; Additional file 1: Table S2). While soil bacteria in Nanjing were significantly enriched in genes associated with xenobiotics biodegradation and metabolism, nucleotide metabolism, folding sorting and degradation, especially for genes involved in biodegradation and metabolism of nitrotoluene, aminobenzoate, naphthalene, steroid and xylene (Additional file 1: Fig. S4). The annual frequency and dosage of chemical pesticide application especially herbicides in Nanjing were higher than those in other two areas (Additional file 1: Table S2). As a result, pesticide residues in the soil were relatively high, and soils were significantly enriched in bacteria with xenobiotics biodegradation genes, presumably to help decompose pesticides in the soil.

**Fig. 5:**
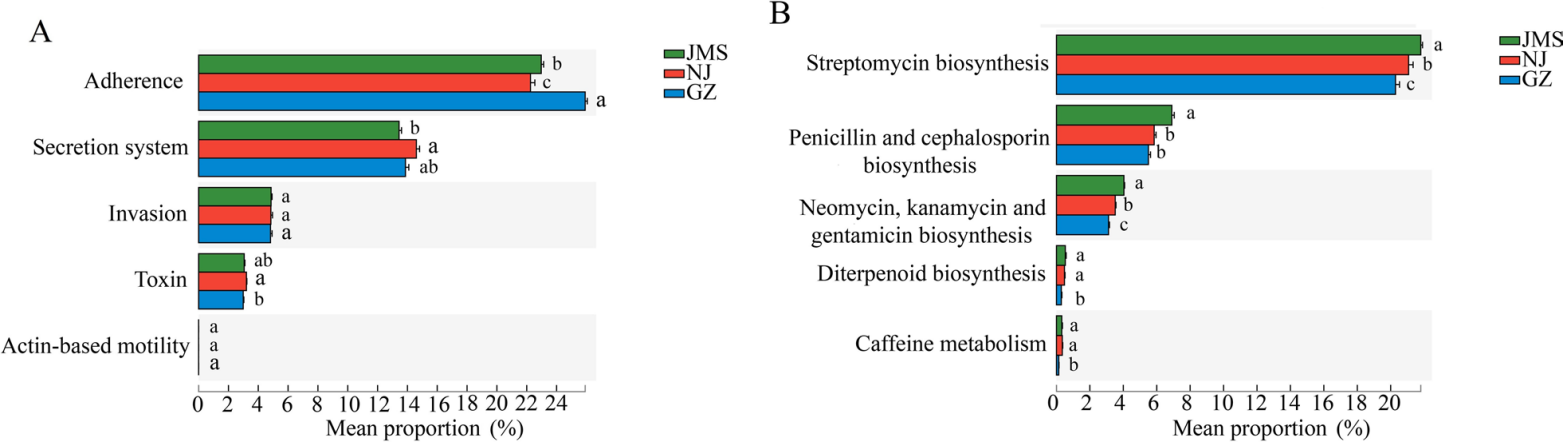
Relative abundance of genes related to virulence factors (**A**) and secondary metabolite biosynthesis (**B**). Different letters above the bars indicate a significant difference (*p* < 0.05) according to Kruskal–Wallis H test. GZ: rice double cropping in Guangzhou; JMS: rice single cropping in Jiamusi; NJ: rice–wheat rotation cropping in Nanjing

Soil bacteria in Guangzhou were significantly enriched in genes associated with bacterial virulence, energy metabolism, translation, cell growth and death, which were positively correlated with annual accumulated temperature and annual precipitation. In addition, the analysis of the virulence factor database VFDB showed that the abundance of genes related to factors (adherence-related genes) in the Guangzhou region was significantly higher than that in Nanjing and Jiamusi, especially for genes for flagella, Trw type IV secretion system, fimbriae, Myf/pH6 antigen, and PI-2, accounting for more than 70% of the total adherence-related genes (Fig. 5C). Functional analysis of biosynthesis of other secondary metabolites showed that the genes related to antimicrobial antibiotic secretion (such as biosynthesis of streptomycin, penicillin and cephalosporin, and neomycin, kanamycin and gentamicin) in Guangzhou were significantly lower compared with that of Nanjing and Jiamusi (Fig. 5D). More serious bacterial diseases in Guangzhou may be related to the higher temperature and humidity and lower secretion of antibiotics, which may be conducive to the reproduction of pathogenic bacteria and to the higher relative abundance of adherence-related genes might aid their infection of the plant host.

### Abundant auxiliary carbohydrate metabolic genes in paddy viruses

**Fig. 6:**
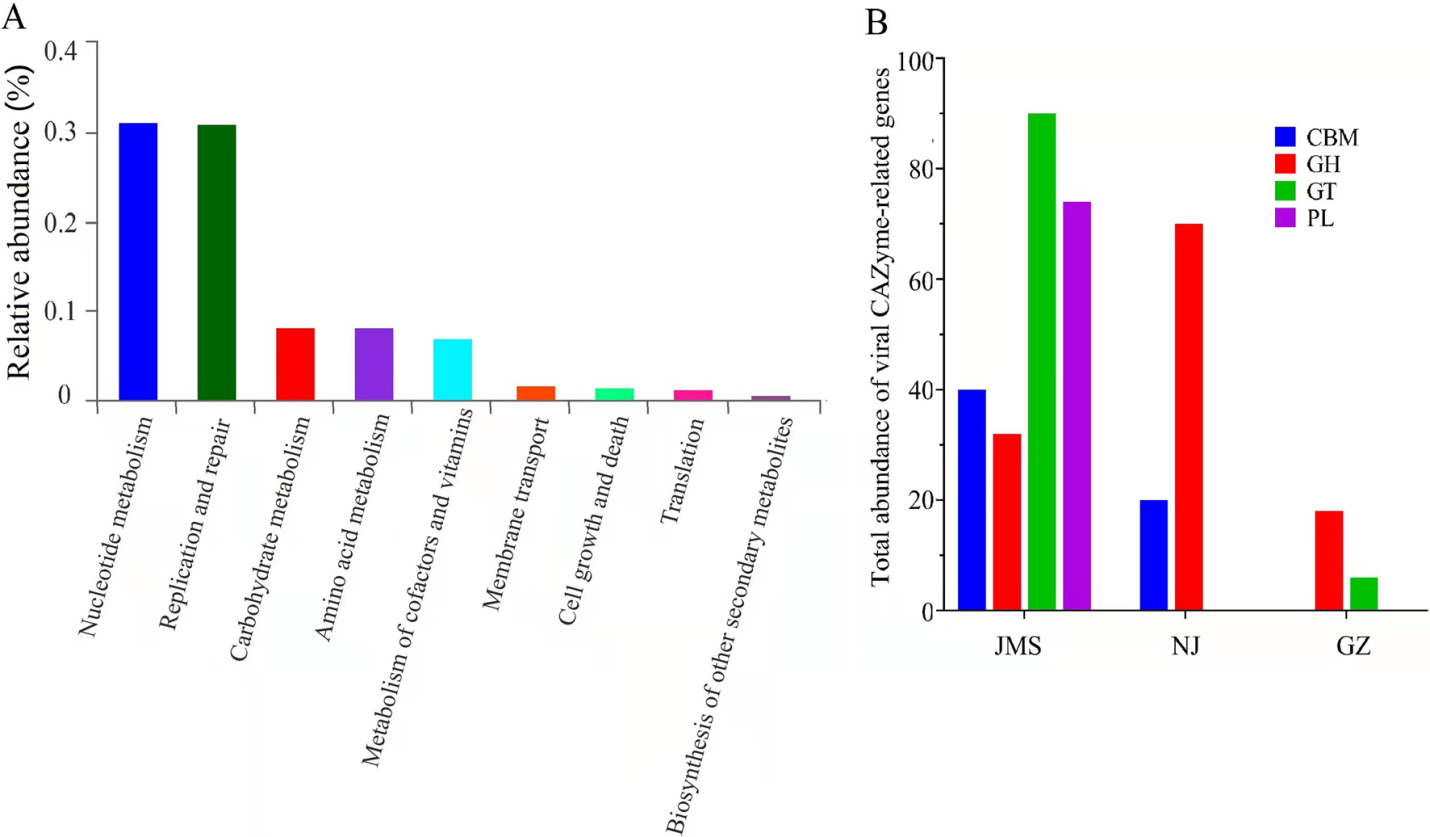
Abundant auxiliary carbohydrate metabolism genes in paddy soil viruses. Functional annotation of paddy soil virome. **A** Functional annotation was predicted with KEGG database (E-value < 1e^−5^). KEGG function class was ordered according to their hit frequency. **B** Annotation of viral carbohydrate metabolism-related ORFs in the CAZy database. GZ: rice double cropping in Guangzhou in blue; JMS: rice single cropping in Jiamusi in red; NJ: rice–wheat rotation cropping in Nanjing. CBM: carbohydrate-binding modules; GH: glycoside hydrolases; GT: glycosyl transferases; PL: polysaccharide lyases

In the functional annotation of viruses in paddy soils using the KEGG database (Fig. 6A), almost all functions in pathway level 2 were found, but the sequences were notably enriched in a limited number of functions; the top three assigned functions accounted for 70% of the annotated sequences. The top two most-abundant functions were nucleotide metabolism, replication and repair, which are critical for the reproduction and survival of viruses. In addition, viruses have genes related to carbohydrate metabolism and the biosynthesis of secondary metabolites; the relative abundances of genes related to carbohydrate metabolism in viruses were negatively correlated (*ρ* = − 0.498, *p* = 0.334) with those in soil bacteria (Additional file 1: Fig. S5A). Similarly, the abundances of genes related to biosynthesis of secondary metabolites in viruses were negatively correlated (*ρ* = − 0.500, *p* = 0.333) with those in soil bacteria (Additional file 1: Fig. S5B). These two types of genes carried by bacteriophages might assist host bacteria in carbon metabolism and secondary metabolite synthesis. In the annotation of carbohydrate-active enzymes (CAZymes) using the CAZy database (Fig. 6B), the species and abundance of viral CAZymes differed significantly among the soils of the three cropping systems. Notably, 67.43% of the CAZymes-related genes were present in Jiamusi, which was 9.83 and 2.62 times higher than in Guangzhou and Nanjing. In addition, there were two classes CAZymes (glycoside hydrolases, glycosyl transferases) in Guangzhou, two classes (glycoside hydrolases, carbohydrate-binding modules) in Nanjing, but four classes (glycoside hydrolases, carbohydrate-binding modules, glycosyl transferases, polysaccharide hydrolase) in Jiamusi. Remarkably, polysaccharide lyases (pectate lyase, exopolygalacturonate lyase, thiopeptidoglycan lyase) were unique to Jiamusi, implying potential roles in the decomposition of soil organic carbon.

### *Enterobacter* bacteriophages can reduce the nitrogen fixation capacity of soil by lysing host bacteria

In Nanjing, the most abundant bacteriophage was *Enterobacter* phage, with a relative abundance of 14.8%, compared with 0.13% in Guangzhou and 0% in Jiamusi (Additional file 1: Fig. S6A). They were also negatively correlated with the abundance of host bacteria *Enterobacter* (*ρ* = − 0.805, *p* = 0.404) (Additional file 1: Fig. S6B). To study its function, the bacteriophage was isolated from the soil in Nanjing and named *Enterobacter* phage-NJ (Additional file 1: Fig. S7A). In the plate lytic experiment, the bacteriophage had lytic activity against nitrogen-fixing *Enterobacter* (Additional file 1: Fig. S7B)*.*

**Fig. 7:**
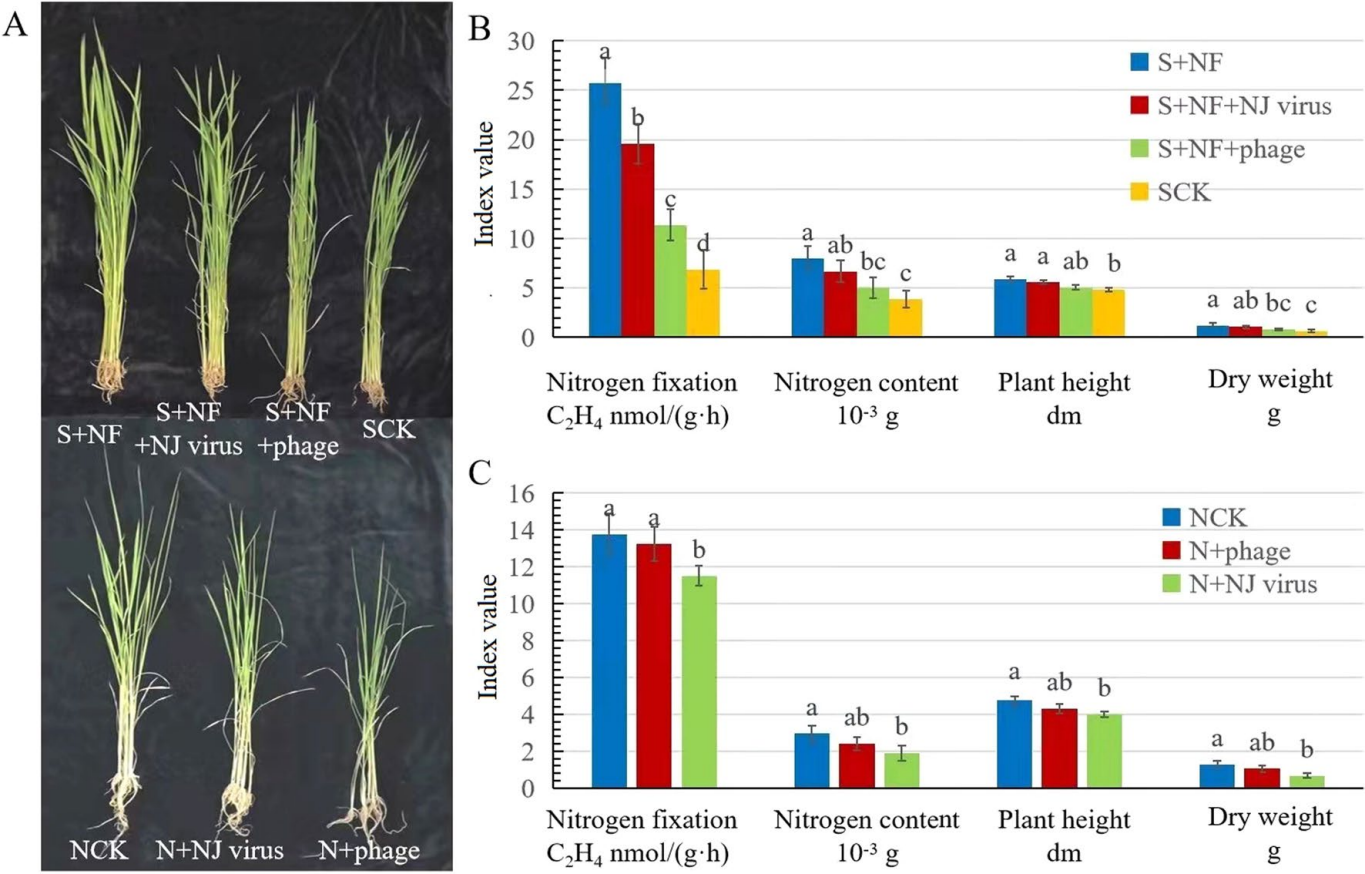
Addition of *Enterobacter* bacteriophage-NJ to soil reduced nitrogen-fixation capacity of *Enterobacter* species and inhibited rice growth. **A** (top row) Representative phenotypes of rice grown in sterile field soil amended with N-fixing *Enterobacter* (S + NF), virus isolated from Nanjing soil and N-fixing *Enterobacter* (S + NF + virus), *Enterobacter* bacteriophage and N-fixing *Enterobacter* (S + NF + phage), or in sterile field soil (SCK) and **B** effects of the treatments on N fixation, rice N content, plant height and dry mass. **A** (bottom row) Representative phenotypes of rice grown in sterile field soil from Nanjing (NCK) or in Nanjing soil amended with virus isolated from Nanjing soil (N + NJ virus) or with *Enterobacter* bacteriophage (N + phage) and **C** effects of treatments on N fixation, rice N content, plant height and dry mass. Different letters above the bars indicate a significant difference (*p* < 0.05) according to Kruskal–Wallis H test

To assess the functional effects of *Enterobacter* phage-NJ on nitrogen fixation and uptake, we added *Enterobacter* phage-NJ to the sterilized soil to which nitrogen-fixing *Enterobacter* (*E*. *cloacae*, *E*. *ancerogenus* and *E*. *ludwigii*) had been added. The results showed that the nitrogen-fixation capacity decreased by 55%, and plant height, fresh mass and nitrogen content of the rice plants were 14.27%, 32.25%, and 37.17%, respectively, lower than in the controls. Similar results were found when Nanjing soil viruses were added to the sterilized soil, the nitrogen-fixation capacity decreased by 23.72%, and plant height, fresh mass and nitrogen content of the rice plants were 5.45%, 12.90% and 16.27%, respectively (Fig. 7A, B). When *Enterobacter* phage-NJ was added to untreated field soil, the nitrogen-fixation capacity decreased by 16.49%, and plant height, fresh mass, and nitrogen content were15.88%, 47.66% and 35.47% respectively, lower than in the controls. Similar results were found when Nanjing soil viruses were added to untreated field soil, the nitrogen-fixation capacity decreased by 3.78%, and plant height, fresh mass, and nitrogen content were 9.29%, 17.97%, and 18.58%, respectively (Fig. 7A, C).

**Fig. 8:**
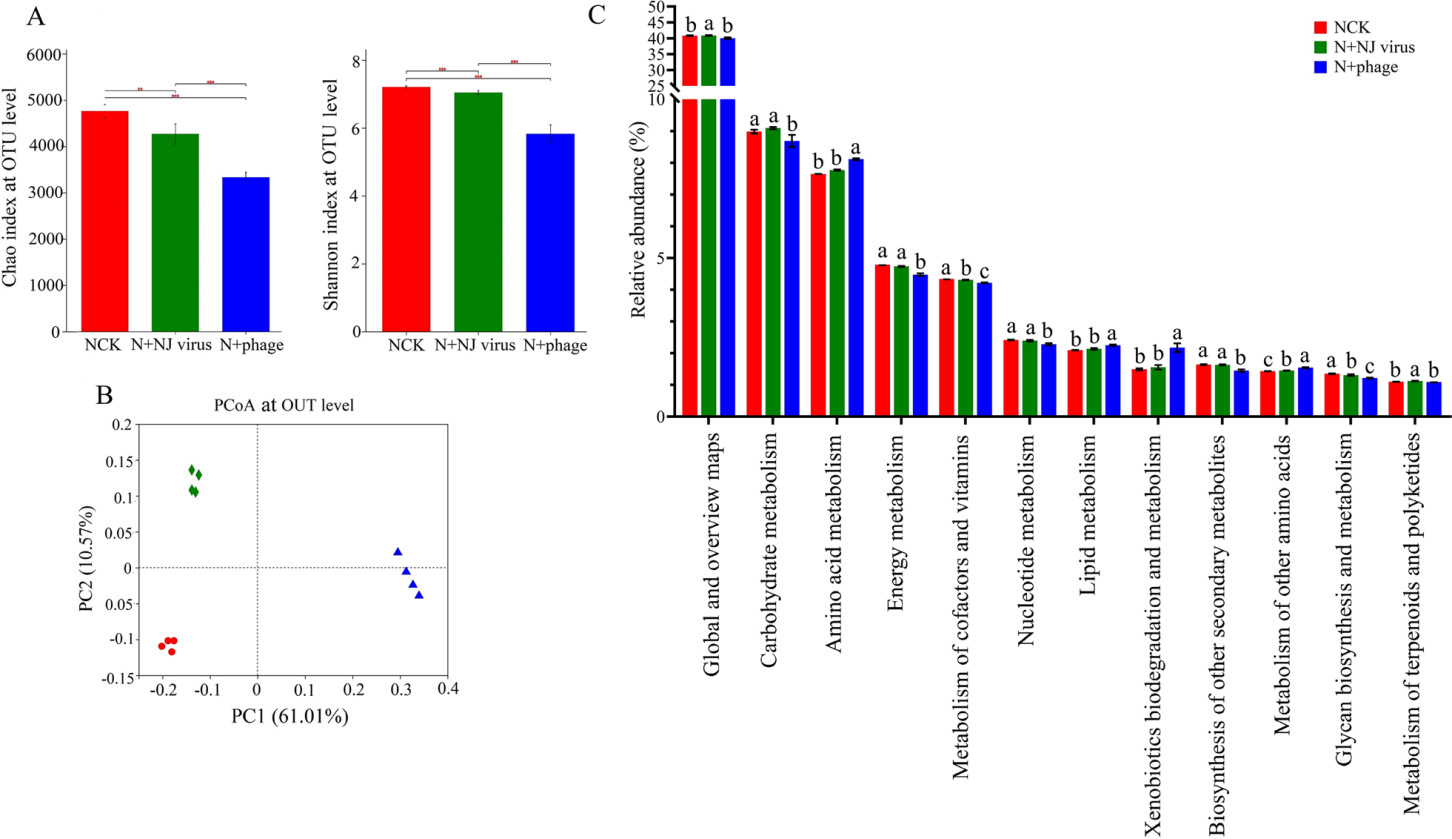
Paddy soil bacterial diversity and function after addition of *Enterobacter* bacteriophage-NJ to untreated field soil from the Nanjing agroecosystem. **A** Variation in alpha diversity of soil bacterial community based on the Chao and the Shannon indices. Values for the same index with different letters above bars differed significantly (*p* < 0.05) according to Kruskal–Wallis H test. **B** Principal coordinate analysis of soil bacterial community based on Bray–Curtis distance matrices. **C** Paddy soil bacterial function after addition of *Enterobacter* bacteriophage-NJ. The *x*- and *y*-axis represent the two selected principal coordinate axes; the percentage represents the explanatory value of the principal coordinate axes. N + NJ virus: virus isolated from NJ soil was added to untreated field soil; N + phage, *Enterobatcter* bacteriophage inoculation in natural soil; NCK: untreated field soil

Rhizosphere bacterial diversity in untreated field soil after the addition of *Enterobacter* phage-NJ was then studied using 16S rRNA amplicon sequencing. After the addition of *Enterobacter* phage-NJ, the richness and diversity of rice rhizosphere microorganisms was 29.97% and 19.17%, respectively, lower than in the control (Fig. 8A). Moreover, the community structure of rhizosphere microorganisms significantly changed; PCoA demonstrated separate clustering (Fig. 8B). In addition, the metabolic function of rhizosphere microorganisms also significantly decreased, especially carbohydrate metabolism, energy metabolism and biosynthesis of secondary metabolites (Fig. 8C).

## Discussion

### Functional differences of soil bacteria in the three rice agroecosystems

Soil bacteria in three rice agroecosystem performed multiple functions simultaneously and were significantly enriched in typical functions that were specific for an agroecosystem. Soil bacteria in Jiamusi and Nanjing were significantly enriched in genes associated with carbohydrate metabolism, xenobiotics biodegradation and metabolism, respectively, while soil bacteria in Guangzhou were enriched in genes associated with virulence factors, but scarce in genes associated with secondary metabolite biosynthesis, which might lead to the significant occurrence of rice bacterial diseases. These significant differences are likely related to the local environment and cropping system. The effects of environment on soil bacterial function can be divided into direct and indirect effects. Environmental factors especially temperature can directly influence the functions by accelerating the activity microbes [27, 28] or indirectly affect functions by altering the composition of microbial communities [29]. Soils in Jiamusi were significantly enriched in genes associated with carbohydrate metabolism, which were positively correlated with the high content of soil organic matter. Soil organic matter can provide nutrients and promote multiplication of microbes, ultimately, indirectly promoting the capacity of carbon metabolism [11]. With lower inputs of plant-derived organic carbon, desert communities have lower relative abundances of genes associated with nutrient cycling and the catabolism of plant-derived organic compounds compared with forests, grasslands, and tundra [30]. Our correlation analysis of environment and function showed that soil bacteria in Nanjing were significantly enriched in genes associated with xenobiotics biodegradation and metabolism, which were positively correlated with the large number of pesticide applications. Because of the rice–wheat rotation system in Nanjing, more types and applications of chemicals are used than in the double cropping system in Guangzhou and in the single cropping rice in Jiamusi. Chemical residues are beneficial to the survival of pesticide-degrading microbes and ultimately result in significant enrichment of the xenobiotics biodegradation and metabolism functions of soil bacteria in Nanjing. The genes associated with bacterial virulence factors in Guangzhou soils were positively correlated with higher annual accumulated temperature and annual precipitation. One of the most important conditions for rice bacterial diseases is high temperature and humidity [31]. In addition, double cropping rice in Guangzhou provides two growing seasons for hosts of bacterial pathogens, so pathogens have more time and opportunity to survive than Nanjing and Jiamusi. These results indicate that functions of soil bacteria vary in different agroecosystems and that environmental factors play important roles through affect microbial community composition.

### Differences in soil viruses among the three agroecosystems and their relationship with host bacteria

In our study, there were significant differences in the species and composition of paddy soil viruses among the three agroecosystems. In Nanjing, 40.64% more virus species were detected than in Jiamusi and 13.54% more than in Guangzhou. A previous study showed that the bacterial host species and abundance may be a key factor controlling viral abundance and distribution [32]. Similarly, we found that soil viral richness was significantly positively correlated with bacterial richness (*ρ* = 0.662, *p* = 0.01). Only 13.39% of the viral species were shared by all three rice agroecosystems, while 59.56% of the viral species were environmentally specific, which is likely associated with the significant differences in temperature, annual precipitation and soil physicochemical properties in the three rice agroecosystems because viral diversity and community structure have been reported to be controlled by factors such as temperature [33], soil moisture [34], and pH [35].

In soils, most viruses are bacteriophages, which can be divided into lysogenic or lytic bacteriophages [15]. The abundance and distribution of bacteriophages and host bacteria influence each other in a complex relationship based on our results. Bacteriophages are obligate intracellular parasites, so their fitness and range limits are influenced by their hosts, and the bacteriophages in turn can affect host abundance and even the community structure and diversity of the whole bacterial community [36–39]. Lytic bacteriophages can modulate host bacterial populations and diversities by lysing their hosts [40], but lysogenic bacteriophages can increase the host abundance by promoting horizontal gene transfer by lysogenic conversion or transferring packaged bacterial genes to new hosts, which may expand the host metabolic profile and enhance microbial environmental adaptability [41]. In our study, we analyzed the relationships between viruses and their potential hosts. When the ratio of viral abundance to bacterial abundance was greater than 100, there was a significant negative correlation between the abundance of bacteriophage and bacteria, indicating that bacteriophages may be lytic, which negatively regulates the abundance of host bacteria. However, when the ratio was less than 100, the correlation between bacteriophage and bacteria abundance was not significant, perhaps because the bacteriophages may be lysogenic, which have little effect on host bacteria abundance, and/or because bacteriophages overlap in their distribution with multiple susceptible hosts in natural environment, while bacteria can often be infected with several different types of bacteriophages. Furthermore, many factors influence the abundance of bacteriophages and host bacteria, making the relationship between them more complex.

### Bacteriophages inhibit soil nitrogen fixation by lysing host bacteria

Lytic bacteriophages have direct and indirect effects on nutrient cycling. They can directly lyse bacteria, which releases organic matter, including nutrients such as carbon, nitrogen and phosphorus. The carbon cycle driven by viruses accounts for 25% of the total carbon cycle in marine agroecosystems [16]. Addition of bacteriophages to soil also increases the NH_4_^+^ concentration via lysis, resulting in a release of inorganic nitrogen, followed by mineralization [42]. Indirectly, bacteriophage lysis can also slow nutrient transformations by reducing the population of key bacteria involved in nutrient cycling [42]. Higher abundance of T4-like phages increases bacterial death, thereby suppressing soil organic carbon mineralization [43]. In wetlands, bacteriophages can infect sulfate-reducing and methanogenic bacteria, which can potentially repress sulfate reduction (and associated carbon mineralization) and methane production, respectively [44]. However, indirect inhibition of biogeochemical cycling by bacteriophages has mostly been inferred by sequencing analysis without relevant experimental verification. In our study, *Enterobacter* phage-NJ, which was isolated from the soil in Nanjing. It can lyse three species *Enterobacter* bacteria which we could get and were proven to have nitrogen-fixing ability by Agricultural Culture Collection of China. When *Enterobacter* phages-NJ were added to untreated field soil or sterilized soil with nitrogen-fixing bacteria, the soil nitrogen-fixation capacity decreased, and rice growth and nitrogen content decreased also. This result provides direct evidence that bacteriophages can inhibit biogeochemical cycling through lysis. *Enterobacter* phage was the most abundant in the tested soils from Nanjing, with a relative abundance of 14.8%. When we extracted all viruses from the Nanjing soils and added them into natural field soil, the result was similar to adding bacteriophages alone; that is, soil nitrogen-fixation capacity was reduced. In addition, the soil bacterial community changed, and the bacterial metabolic functions such carbon metabolism and energy metabolism decreased also. These results indicate that bacteriophages can regulate soil bacterial community structure and ecological function and that the differences in soil bacterial function were related to differences in the bacteriophages in the different agroecosystems.

### Potential effects of auxiliary metabolic genes of phages on soil microbial function

Bacteriophages can affect nutrient cycling not only by lysing the host but also by metabolically reprogramming their hosts via the horizontal transfer of ecologically important genes and the expression of virus-carried auxiliary metabolic genes (AMGs) [45]. AMGs have been most extensively explored in marine ecosystem and are involved in photosynthesis, carbon metabolism, nitrogen metabolism and phosphate uptake [46, 47]. Those genes are hypothesized to increase viral survival and replication by augmenting key steps in host metabolism. In mangrove soil [24], permafrost soil [48], and maize–barley rotation soil [20], AMGs involved in carbohydrate metabolism are notably over-represented. In the maize–barley rotation soil, three classes of genes—carbohydrate-binding modules, carbohydrate esterases, glycoside hydrolases—were identified. Similarly, in paddy soil, four classes of AMGs of bacteriophages involved in carbon metabolism were identified in our study (glycoside hydrolases, glycosyl transferases, polysaccharide lyases, and carbohydrate-binding modules, with the abundance of glycoside hydrolases the highest), indicating that paddy soil viruses primarily participate in the decomposition of organic carbon, breakdown of complex carbohydrates to increase energy production and boost bacteriophage replication. The species and abundance of AMGs also differed among the three agroecosystems we studied. Compared with the bacteriophages in the other two agroecosystems, the bacteriophages in Jiamusi had the greatest diversity of AMGs genes related to carbon metabolism; there were four classes of CAZymes in Jiamusi (polysaccharide lyases, glycoside hydrolases, glycosyl transferases, carbohydrate-binding modules), but only two in Guangzhou (glycoside hydrolases, glycosyl transferases) and in Nanjing (glycoside hydrolases, carbohydrate-binding modules). However, soil bacteria in Jiamusi were significantly enriched for carbon metabolism function, likely due to the large diversity of bacteriophage AMGs genes related to carbon metabolism. Remarkably, polysaccharide lyases (pectate lyase, exopolygalacturonate lyase, thiopeptidoglycan lyase) were unique to Jiamusi. During the infection cycle, bacteriophages need to break down the host bacterial surface barriers such as capsule polysaccharides (CPSs), extracellular polysaccharides (EPSs). Polysaccharide lyases carried by bacteriophages can help to break down the barrier and infect the host [49]. In addition, complex carbohydrates or polysaccharides such as cellulose, xylan, pectin, starch, alginate, mannan, and chitin are major components of plant cell walls, which are difficult to degrade. In Jiamusi, the soil organic matter content is the highest, perhaps because the bacteriophages carry genes for polysaccharide lyases, which might assist host bacteria in degrading organic matter in the soil.

## Conclusion

In our analyses to identify and compare the bacterial and viral communities in soils from the three most important rice agroecosystems in China, the communities in Jiamusi were significantly enriched in genes related to carbohydrate metabolism, in Nanjing with xenobiotics biodegradation and metabolism, and in Guangzhou with genes associated with virulence factors, but those involved with secondary metabolite biosynthesis were scarce, which might lead to a significant incidence of rice bacterial diseases. The 368 virus species that were detected from the three agroecosystems likely affect the soil microbial community structure and function. Bacteriophage isolation and inoculation experiments showed that *Enterobacter* phage-NJ not only changed the soil bacterial diversity and community structure, but also reduced the nitrogen-fixation capacity by lysing nitrogen-fixing host bacteria. In addition, auxiliary carbohydrate-active enzyme (CAZyme) genes were notably over-represented in soil viruses, and 67.43% of all CAZyme genes were present in Jiamusi soils, which might assist host bacteria in metabolizing carbon. Our study will contribute to a better understanding of the microbial diversity in the typical rice agroecosystems in China, viral mechanisms that may be involved in this diversity and inform management programs for the safe production of healthy rice.

## Methods

### Sampling sites and sample collection

Paddy soil samples were collected during heading of rice in 2019 from long-term (more than 20 years) rice farms in Jiamusi (46° 48′ N, 130° 22′ E, 130 m a.s.l.) in Heilongjiang Province, Nanjing (30° 02′ N, 118° 46′ E, 30 m a.s.l.) in Jiangsu Province, and Guangzhou (23° 13′ N, 113.27′ E, 871 m a.s.l.) in Guangdong Province in China. In Jiamusi, Nanjing and Guangzhou, the annual precipitation is 510 mm, 1090 mm and 1720 mm, respectively, with 67%, 73%, and 77% mean humidity, respectively, and the annual accumulated temperature of 2700 °C, 5400 °C and 7500 °C, respectively (http://www.weather.com.cn). The three regions different in their rice planting systems (single rice cropping in Jiamusi, rice–wheat rotation in Nanjing, double rice cropping in Guangzhou). Rice was grown at each farm using local practices for cultivation, fertilizer application, and field management. Chemical pesticides were applied as shown in Additional file 1: Table S1. Four plots (5 × 10 m) were randomly selected for collecting 30 cores of bulk soil samples (0–15 cm) at each farm. At the same time, plants were inspected for naturally occurring diseases in four randomly chosen, representative 5-m lengths of rows from each plot. The severity of rice leaf diseases (rice brown spot, brown streak, bacterial blight, bacterial leaf streak and foot rot) was visually scored on a 0 to 5 scale based on the estimated percentage leaf area with lesions: 0, no symptoms; 1, 0.1–6% of total leaf area; 2, 7–12%; 3, 13–25%; 4, 26–50%; 5, 51–100% [50]. A disease severity index was then calculated as ∑ (Number of diseased plants with each score × Highest score)/(Total number of plants × Highest score) × 100. Disease incidence of rice bacterial foot rot was investigated as the (Number of plants with blackened, rotting roots/Total number of examined plants) × 100. Soil samples were transported to the laboratory on ice in a cooler. A portion of the soil sample was stored at − 80 °C for DNA extraction, and the remainder was stored at 4 °C for analyses of soil chemical properties.

### Analyses of soil chemical properties

Soil pH was determined using a Mettler-Toledo FE20-Five Easy PlusTM pH meter (Schwerzenbach, Switzerland) in a 1:5 soil–water (w/v) suspension. Soil organic carbon was measured using the chromic acid titration method [51]. Soil available nitrogen was determined by the alkali diffusion method, soil available phosphorus was determined using the sodium hydrogen carbonate solution-Mo-Sb anti spectrophotometric method, soil available potassium was measured by the ammonium acetate method, followed by flame photometric detection [52].

### DNA extraction

Microbial genomic DNA was extracted from 0.5 g soil with the E.Z.N.A. Soil DNA Kit (Omega Bio-TEK, Norcross, GA, USA) according to the manufacturer’s instructions. Concentration and purity of extracted DNA were determined with a TBS-380 Mini-Fluorometer (Turner Biosystems, CA, USA) and NanoDrop2000 UV–Vis spectrophotometer (Thermo Scientific, Wilmington, DE, USA), respectively. DNA quality was confirmed using 1.2% agarose gel electrophoresis, with 1 × TAE buffer (40 mM Tris–HCl, 40 mM acetate, 1.0 mM EDTA) and ethidium bromide (0.5 µg mL^−1^) under ultraviolet light [53].

### Shotgun metagenomic analysis

The extracted DNA was fragmented to an average size of about 400 bp using Covaris M220 (Gene Company Limited, Hong Kong, China). A paired-end library was constructed using NEXTFLEX® Rapid DNA-Seq (Bioo Scientific, Austin, TX, USA). Paired-end sequencing was performed using the Illumina HiSeq 4000 platform (Illumina Inc., San Diego, CA, USA) at Majorbio Bio-Pharm Technology Co., Ltd. (Shanghai, China), HiSeq 3000/4000 PE Cluster Kit and HiSeq 3000/4000 SBS Kit according to the manufacturer’s instructions (www.illumina.com). Sequence data associated with this project were deposited in the NCBI Short Read Archive database (accession PRJNA732820). The paired-end Illumina reads were trimmed of adaptors, and low-quality reads (length < 50 bp or with a quality value < 20 or having N bases) were removed by fastp version 0.20.0 (https://github.com/OpenGene/fastp). Metagenomics data were assembled using MEGAHIT version 1.1.2 (https://github.com/voutcn/megahit). Contigs of ≥ 300 bp were selected for further gene prediction and annotation. BLASTP against the NCBI NR database was used for taxonomic annotations of the representative sequences with an e-value cut-off 1e^−5^ using Diamond (http://www.diamondsearch.org/index.php, version 0.8.35). The KEGG annotation was conducted using Diamond against the Kyoto Encyclopedia of Genes and Genomes database (KEGG) version 92.0 (http://www.genome.jp/keeg/) with an e-value cutoff of 1e^−5^. Virulence factors were annotated using the Virulence Factors Database (VFDB) database version 2016.03 (http://www.mgc.ac.cn/VFs/main.htm) with an e-value cutoff of 1e^−5^. Carbohydrate-active enzymes were identified using HMMscan tools and the CarbohydrateActive enZYmes Database (CAZy) version 6.0 (http://www.cazy.org/) with an e-value cutoff of 1e^−5^. We also selected the bacterial and viral genes and built bacterial and viral sets for taxonomic, KEGG and CAZY annotations. Clustered Regularly Interspaced Short Palindromic Repeats (CRISPR) were used to predict phage host based on the sequence similarity between the spacers in microbial CRISPR regions and the protospacers in viral genomes [54].

### Isolation of soil viruses

Soil sample (1 kg) was suspended in 1 L of SM solution (100 mM NaCl, 8 mM MgSO_4_·7H_2_O and 50 mM Tris/HCl, pH 7.5) and shaken for 2 h at 30 °C. The soil particles were removed by centrifugation at 4000×*g* for 15 min. The supernatant was filtered sequentially through 0.45 and 0.22 μm filters. Then, virus particles in the filtered supernatant were concentrated into 1 mL by centrifugation at 4000×*g* in 100 kDa centrifugal ultrafiltration tubes.

### Isolation of *Enterobacter* bacteriophage from paddy soil

Soil sample (10 g) was suspended in 50 mL Luria Bertani (LB) broth (1% tryptone, 0.5% yeast extract, and 1% NaCl) and dispersed by shaking for 12 h at 30 °C. The soil particles were removed by centrifugation at 4000×*g* for 15 min, and the supernatant was filter-sterilized through a 0.22 μm filter. One milliliter of filtered supernatant was added to 10 mL of an overnight suspension culture of *Enterobacter cloacae* (proven to be nitrogen-fixing and provided by Agricultural Culture Collection of China) and incubated for 12 h at 30 °C [55]. Then bacterial cells were removed by centrifugation at 4000×*g* for 15 min, and the supernatant was sterilized through a 0.22 μm filter. Then 0.1 mL of the filtered supernatant was mixed with 1 mL of the *E. cloacae* suspension (OD600 = 1). This mixture was then added to 2.5 mL of soft agar (0.35% agar prepared in 1% tryptone, 0.5% NaCl, 3 mM MgCl_2_, 3 mM CaCl_2_, and 0.04% [wt / vol] glucose) and poured in a uniform layer onto an LB agar plate. Bacteriophage plaques were identified after an overnight incubation at 30 °C. Bacteriophages were purified via five consecutive transfers of them from individual plaques to new bacterial cell lawns. The purified bacteriophage titer was determined by mixing 100 μL of host cells (OD600 = 1) and 100 μL of bacteriophage dilution with soft agar and pouring the mixture over LB agar in a plate. Plaques were scored after an overnight incubation at 30 °C. Bacteriophages were kept in buffer (10 mM Tris [pH 7.6], 5 mM MgSO_4_·7H_2_O, 0.01% gelatin) at 4 °C.

Each isolated *Enterobacter* phage was analyzed for its ability to lyse *E. ancerogenus* and *E. ludwigii* (proven to be nitrogen-fixing and provided by Agricultural Culture Collection of China) using a plate lytic experiment with 0.1 mL bacteriophage mixed with 1 mL of overnight-cultured *Enterobacter* strain and shaken (200 rpm) at room temperature for 20 min, then 3 mL of soft nutrient agar was added, and the mixture was poured on LB agar plates and incubated overnight at 30 °C. Lytic activity was indicated by transparent plaques [56].

### Impact of *Enterobacter* bacteriophages on soil nitrogen-fixation capacity

In a greenhouse experiment, the influences of *Enterobacter* phages on soil nitrogen fixation capacity in untreated field soil and in sterilized field soil were investigated. Rice seeds were surface-sterilized and pregerminated, then seedlings with a primary root length of 1–2 cm were planted in pots (6 seeds per pot) with the untreated field soil. Three treatments with four pots (each pot filled with 1 kg soil) were set up: (1) N + NJ virus, 1 mL virus isolated from 1 kg Nanjing soil was diluted to 50 mL with ddH_2_O and added to each pot after planting rice; (2) N + phage, 1 mL *Enterobacter* bacteriophages (10^7^ PFUs) diluted to 50 mL with ddH_2_O was added after planting rice; (3) NCK, 50 mL ddH_2_O was added as control. After treatment, rice plants were grown in a greenhouse at 26 °C with 14 h of fluorescent light and 10 h of darkness for 7 weeks and watered with 20 mL ddH_2_O every 3 days.

Untreated field soil was autoclaved using the liquid cycle for 30 min at 121 °C, then cooled at room temperature, then we repeated this procedure, sterilized soil was stored at 4 °C until use. Mixed *Enterobacter* species (*E. cloacae-*211, *E. ancerogenus-*212, *E. ludwigii*-213, ratio 1:1:1) were cultured in LB broth overnight at 30 °C, followed by centrifugation at 1000×*g* for 5 min. The pellet was resuspended in ddH_2_O and diluted to an optical density OD600 of 0.02. Rice seeds were surface-sterilized and pregerminated, then seedlings with primary root length of 1–2 cm) were planted in pots (6 seeds per pot) with sterilized soil. Four treatments were set up with four pots per treatment: (1) S + NF, 50 mL *Enterobacter* strains were added after rice planting, then cultured for a week and added with ddH_2_O; (2) S + NF + virus, 50 mL *Enterobacter* strains were added after rice planting, then cultured for a week and added with 1 mL virus isolated from 1 kg Nanjing soil, which was diluted to 50 mL with ddH_2_O; (3) S + NF + phage, 50 mL *Enterobacter* strains were added after rice planting, then cultured for a week and watered with 1 mL *Enterobacter* bacteriophages (10^7^ PFUs) was diluted to 50 mL with ddH_2_O; (4) SCK, sterile water was used as the control. After treatment, rice plants were grown in a greenhouse at 26 °C with 14 h of fluorescent light and 10 h of darkness for 7 weeks and watered with 20 mL ddH_2_O every 3 days. After 7 weeks, rhizosphere soil samples were collected [57], rice height and fresh mass were measured. Then rice plants were dried and grounded, nitrogen concentrations were determined with a modified Kjeldahl digestion method [58]. N_2_ fixation in soil samples was assessed indirectly using the acetylene reduction assay measuring ethylene formation from acetylene [59].

### Illumina MiSeq sequencing

Region V3-V4 of the bacterial 16S rRNA gene was amplified from each sample using primers 338F (5′-ACTCCTACGGGAGGCAGCAG-3′) and 806R (5′-GGACTACHVGGGTWTCTAAT-3′) in a thermocycler PCR system (GeneAmp 9700, ABI, Foster, CA, United States); barcode was added before the primer to build different libraries in the same sequencing pool. The PCR mixture contained 4 μL 5 × TransStart FastPfu buffer, 2 μL 2.5 mM dNTPs, 0.8 μL 5 μM forward primer, 0.8 μL 5 μM reverse primer, 0.4 μL TransStart FastPfu DNA Polymerase, 10 ng template DNA, with ddH_2_O added to reach 20 μL. PCR cycling conditions were initial denaturation at 95 °C for 3 min; 35 cycles of denaturing at 95 °C for 30 s, annealing at 55 °C for 30 s and extension at 72 °C for 45 s; and single extension at 72 °C for 10 min, and end at 4 °C. The PCR product was extracted from 2% agarose gel and purified using the AxyPrep DNA Gel Extraction Kit (Axygen Biosciences, Union City, CA, USA), then quantified using a Quantus Fluorometer (Promega, Madison, WI, USA). Purified amplicons were paired-end sequenced (2 × 300) on an Illumina MiSeq platform (Illumina, San Diego, CA, USA) according to standard protocols by Majorbio Bio-Pharm Technology Co. Ltd. (Shanghai, China). The raw reads were deposited in the NCBI Sequence Read Archive (SRA) database (accession No. PRJNA780198).

The raw sequencing reads were demultiplexed, quality-filtered by Trimmomatic and merged by FLASH [60]. Operational taxonomic units (OTUs) with 97% similarity cutoff were clustered using UPARSE version 7.1 (http://drive5.com/uparse/), and chimeric sequences were identified and removed. The abundance of OTUs was rarefied to the lowest number of shared OTUs to remove the effect of sequencing depth across samples. The taxonomy of each 16S gene sequence was analyzed using the UNITE version7.2 (http://unite.ut.ee) database with a confidence threshold of 70% [61].

### Statistical analyses

Shannon and Chao indices were calculated by Mothur version v.1.30 (http://www.mothur.org/) with the vegan package to compare the alpha diversity of soil microbial communities. Principal coordinate analysis (PCoA) was performed in R version 3.0.2 (https://www.r-project.org/), R Core package using Bray–Curtis distances to compare soil microbial communities. Circos figures were obtained from Circos-0.67–7 (http://circos.ca/) and reflected the proportion of prominent genera in each sample [62]. Redundancy analysis (RDA) was executed in R version 3.0.2 to analyze the relationship between dominant (abundance > 1%) genera or function at KEGG level 2 and environmental factors. Viral genes of virus were picked out to create a viral gene set to analyze the relative abundance of viruses and its functions in soil virus. Spearman’s correlation between virus and bacteria or microbial function and environmental factors were calculated using the rcorr function in the R package “Hmisc” (v. 4.0-3). Data normally distributed were tested for significant differences with Tukey test after a one-way analysis of variance (ANOVA); data not normally distributed were tested with nonparametric Kruskal–Wallis test using SPSS version 16.0 (SPSS, Chicago, IL, USA). Figures were drawn in R version 3.0.2.

## Supplementary Information

**Additional file 1.** **Table S1.** Chemical applications (apps.) on farms using three rice cropping systems. GZ: Rice double cropping ecosystems in Guangzhou; JMS: Rice single cropping ecosystems in Jiamusi; NJ: Rice-wheat rotation cropping ecosystems in Nanjing. **Table S2.** Means ± standard deviation for environmental factors variables at the three agroecosystem sites in China. **Table S3.** Percentage distribution of the dominant genera in soils of three rice cropping agroecosystems. GZ: rice double cropping in Guangzhou; JMS: rice single cropping in Jiamusi; NJ: rice–wheat rotation in Nanjing. **Fig. S1.** Relative abundance of main microbial phyla (A) and genera (B) in soils of three rice cropping agroecosystems. GZ: rice double cropping in Guangzhou; JMS: rice single cropping in Jiamusi; NJ: rice–wheat rotation in Nanjing. **Fig. S2.** Principal coordinate analysis (PCoA) of soil microbial community based on Bray–Curtis distance matrices. The percentage represents the explanatory value of the PCoA axes to the difference in sample composition; distances between symbols on the ordination plot reflect relative dissimilarities. GZ: rice double cropping in Guangzhou; JMS: rice single cropping in Jiamusi; NJ: rice–wheat rotation cropping in Nanjing. **Fig. S3.** Relative abundance of potentially pathogenic bacteria (A) and disease index for four rice diseases in the three agroecosystems in China. Different letters above the bars indicate a significant difference (*p* < 0.05) according to Kruskal-Wallis H test. GZ: rice double cropping in Guangzhou; JMS: rice single cropping in Jiamusi; NJ: rice–wheat rotation cropping in Nanjing. **Fig. S4.** Relative abundance of genes related to xenobiotics biodegradation and metabolism in viruses and bacteria in soils of three rice agroecosystems. GZ: rice double cropping in Guangzhou; JMS: rice single cropping in Jiamusi; NJ: rice–wheat rotation in Nanjing. Different letters above the bars indicate a significant difference (*p* < 0.05) according to Kruskal-Wallis H test. **Fig. S5.** Relative abundance of genes related to carbohydrate (CHO) metabolism (A) and biosynthesis of secondary (Sec) metabolites (B) in viruses and bacteria in soils of three rice agroecosystems. GZ: rice double cropping in Guangzhou; JMS: rice single cropping in Jiamusi; NJ: rice–wheat rotation in Nanjing. Different letters above the bars indicate a significant difference (*p* < 0.05) according to Kruskal-Wallis H test. **Fig. S6.** Relative abundance of *Enterboacter* phage among soil viruses (A) and relative abundance of *Enterboacter* among soil bacteria (B) in the three rice agroecosystems. GZ: rice double cropping in Guangzhou; JMS: rice single cropping in Jiamusi; NJ: rice–wheat rotation in Nanjing. Different letters above the bars indicate a significant difference (*p* < 0.05)according to Kruskal-Wallis H test. **Fig. S7.** (A) Transmission electron micrograph of *Enterobacter* phage-NJ. (B) Plate lytic experiment of *Enterobacter* phage-NJ against nitrogen-fixing *Enterobacter cloacae*.

## Data Availability

All the raw sequence data for metagenomic sequencing were submitted to the NCBI Sequence Read Archive (http://www.ncbi.nlm.) database under accession number PRJNA732820 and PRJNA780198.
