# Supplementary material for: Heterogeneity of soil bacterial and bacteriophage communities in three rice agroecosystems and potential impacts of bacteriophage on nutrient cycling
Source: Environ Microbiome. 2022 Apr 6;17:17. doi: 10.1186/s40793-022-00410-8 (PMC8985318; doi:10.1186/s40793-022-00410-8)
Supplement: Supplementary file 1 — Additional file 1. Table S1. Chemical applications (apps.) on farms using three rice cropping systems. GZ: Rice double cropping ecosystems in Guangzhou; JMS: Rice single cropping ecosystems in Jiamusi; NJ: Rice-wheat rotation cropping ecosystems in Nanjing. Table S2. Means ± standard deviation for environmental factors variables at the three agroecosystem sites in China. Table S3. Percentage distribution of the dominant genera in soils of three rice cropping agroecosystems. GZ: rice double cropping in Guangzhou; JMS: rice single cropping in Jiamusi; NJ: rice–wheat rotation in Nanjing. Fig. S1. Relative abundance of main microbial phyla (A) and genera (B) in soils of three rice cropping agroecosystems. GZ: rice double cropping in Guangzhou; JMS: rice single cropping in Jiamusi; NJ: rice–wheat rotation in Nanjing. Fig. S2. Principal coordinate analysis (PCoA) of soil microbial community based on Bray–Curtis distance matrices. The percentage represents the explanatory value of the PCoA axes to the difference in sample composition; distances between symbols on the ordination plot reflect relative dissimilarities. GZ: rice double cropping in Guangzhou; JMS: rice single cropping in Jiamusi; NJ: rice–wheat rotation cropping in Nanjing. Fig. S3. Relative abundance of potentially pathogenic bacteria (A) and disease index for four rice diseases in the three agroecosystems in China. Different letters above the bars indicate a significant difference (p < 0.05) according to Kruskal-Wallis H test. GZ: rice double cropping in Guangzhou; JMS: rice single cropping in Jiamusi; NJ: rice–wheat rotation cropping in Nanjing. Fig. S4. Relative abundance of genes related to xenobiotics biodegradation and metabolism in viruses and bacteria in soils of three rice agroecosystems. GZ: rice double cropping in Guangzhou; JMS: rice single cropping in Jiamusi; NJ: rice–wheat rotation in Nanjing. Different letters above the bars indicate a significant difference (p < 0.05) according to Kruskal-Wall [file 40793_2022_410_MOESM1_ESM.doc]

**Heterogeneity of soil bacterial and bacteriophage communities in three rice agroecosystems and potential impacts of bacteriophage on nutrient cycling**

Yajiao Wang^1,2^, Yu Liu^1^, Yuxing Wu^2^, Nan Wu^1^, Wenwen Liu^1^, Xifeng Wang^1^

^1^ State Key Laboratory for Biology of Plant Diseases and Insect Pests, Institute of Plant Protection, Chinese Academy of Agricultural Sciences, Beijing 100193, China

^2^ Institute of Plant Protection, Hebei Academy of Agricultural and Forestry Sciences, Baoding 071000, China

*Correspondence authors: Xifeng Wang ([wangxifeng@caas.cn](mailto:wangxifeng@caas.cn))

Table S1 Chemical applications (apps.) on farms using three rice cropping systems. GZ: Rice double cropping ecosystems in Guangzhou; JMS: Rice single cropping ecosystems in Jiamusi; NJ: Rice-wheat rotation cropping ecosystems in Nanjing.

| [System](../../../../C:/Users/%25E6%259D%258E%25E6%25B3%25A213716587429/AppData/Local/youdao/dict/Application/8.9.9.0/resultui/html/index.html#/javascript:;) | Crop | Type of pesticide | Chemical | Growth period of app. | Dose /growing season (g/hectare) |
| --- | --- | --- | --- | --- | --- |
| GZ | Rice (double cropping / year) | insecticide | Imidacloprid | seedling | 30 |
|  |  |  | Thiamethoxam+lambda-cyhalothrin | tillering | 22.5 |
|  |  |  | Abamectin | heading | 6 |
|  |  | fungicide | Isoprothiolane | tillering | 600 |
|  |  | bactericide | Bismerthiazol | 3 leaf stage | 375 |
|  |  | herbicide | Bensulfuron methyl | sowing | 30 |
| JMS | Rice (single cropping / year) | insecticide | Thiamethoxam+lambda-cyhalothrin | heading | 22.5 |
|  |  | fungicide | Fludioxonil | seed pelleting | 6 |
|  |  |  | Isoprothiolane | heading | 600 |
|  |  | herbicide | Bensulfuron methyl | sowing | 180 |
|  |  |  | Pyrazosulfuron-ethyl | 3 leaf stage | 60 |
| NJ | Rice (single cropping / year) | insecticide | Imidacloprid | tillering | 60 |
|  |  |  | Thiamethoxam+lambda-cyhalothrin | heading | 22.5 |
|  |  | fungicide | Isoprothiolane | tillering | 600 |
|  |  |  | Pyraclostrobin | heading | 90 |
|  |  | herbicide | Bensulfuron methyl | sowing | 180 |
|  |  |  | Pyrazosulfuron-ethyl | 3 leaf stage | 30 |
|  | Wheat (single cropping / year) | insecticide | Imidacloprid | seed pelleting | 22.5 |
|  |  |  | Lambda-cyhalothrin | reviving | 15 |
|  |  | fungicide | Fludioxonil | seed pelleting | 6 |
|  |  |  | Carbendazim | reviving | 600 |
|  |  |  | Tebuconazole | flowering | 75 |
|  |  | herbicide | Bensulfuron-methyl | sowing | 30 |
|  |  |  | Tribenuron-methyl+bensulfuron methyl | reviving | 45 |
|  |  |  |  |  |  |

Table S2 Means ± standard deviation for environmental factors variables at the three agroecosystem sites in China.

| [System](../../../../C:/Users/%25E6%259D%258E%25E6%25B3%25A213716587429/AppData/Local/youdao/dict/Application/8.9.9.0/resultui/html/index.html#/javascript:;) | AK (mg/kg) | AN (mg/kg) | SOC (g/kg) | AP (mg/kg) | AAT (℃) | AP (mm) |
| --- | --- | --- | --- | --- | --- | --- |
| JMS | 121.51 ± 62.03 a | 75.25 ± 1.43 a | 5.032 ± 0.13 a | 108.44 ± 4.81 b | 3200 | 518 |
| GZ | 41.778 ± 1.28 c | 57.167 ± 1.65 c | 3.038 ± 0.19 b | 247.61 ± 4.15 a | 7500 | 1800 |
| NJ | 62.287 ± 1.56 b | 64.167 ± 3.30 b | 3.147 ± 0.08 b | 64.78 ± 1.20 c | 5400 | 1047 |

Different lowercase letters within a column indicate a significant difference in a variable among sites (*P* < 0.05) according to Tukey’s test. GZ: rice double cropping in Guangzhou; JMS: rice single cropping in Jiamusi; NJ: rice–wheat rotation cropping in Nanjing; SOC: soil organic carbon; AK: available potassium, AN: available nitrogen; AP: available phosphorus; AAT: annual accumulated temperature; AP: annual precipitation.

Table S3 [Percentage distribution](../../../../C:/Program%20Files%20(x86)/Youdao/Dict/8.9.3.0/resultui/html/index.html#/javascript:;) of the dominant genera in soils of three rice cropping agroecosystems. GZ: rice double cropping in Guangzhou; JMS: rice single cropping in Jiamusi; NJ: rice–wheat rotation in Nanjing.

| Genus | GZ (%) | JMS (%) | NJ (%) |
| --- | --- | --- | --- |
| *Nocardioides* | 0.86 | 0.74 | 3.38 |
| *Gemmatimonas* | 2.16 | 1.21 | 2.53 |
| *Solirubrobacter* | 0.94 | 0.65 | 2.43 |
| *Conexibacter* | 0.89 | 0.50 | 2.43 |
| *Mycobacterium* | 1.28 | 0.44 | 2.40 |
| *Gemmatirosa* | 1.17 | 0.86 | 2.14 |
| *Streptomyces* | 1.13 | 1.14 | 1.98 |
| *Phycicoccus* | 0.32 | 0.77 | 1.91 |
| *Sphingomonas* | 0.61 | 0.59 | 1.52 |
| *Candidatus_Solibacter* | 1.64 | 2.43 | 1.49 |
| *Bradyrhizobium* | 1.28 | 2.62 | 1.44 |
| *Rhodoplanes* | 0.89 | 0.44 | 1.10 |
| *Pseudolabrys* | 1.31 | 0.14 | 1.04 |
| *Anaeromyxobacter* | 2.04 | 2.36 | 0.88 |
| *Anaerolinea* | 1.37 | 1.85 | 0.84 |
| *Ardenticatena* | 0.75 | 1.19 | 0.61 |
| *Longilinea* | 0.86 | 1.38 | 0.53 |
| *Levilinea* | 0.77 | 1.21 | 0.46 |
| *Nitrospira* | 1.11 | 0.27 | 0.47 |


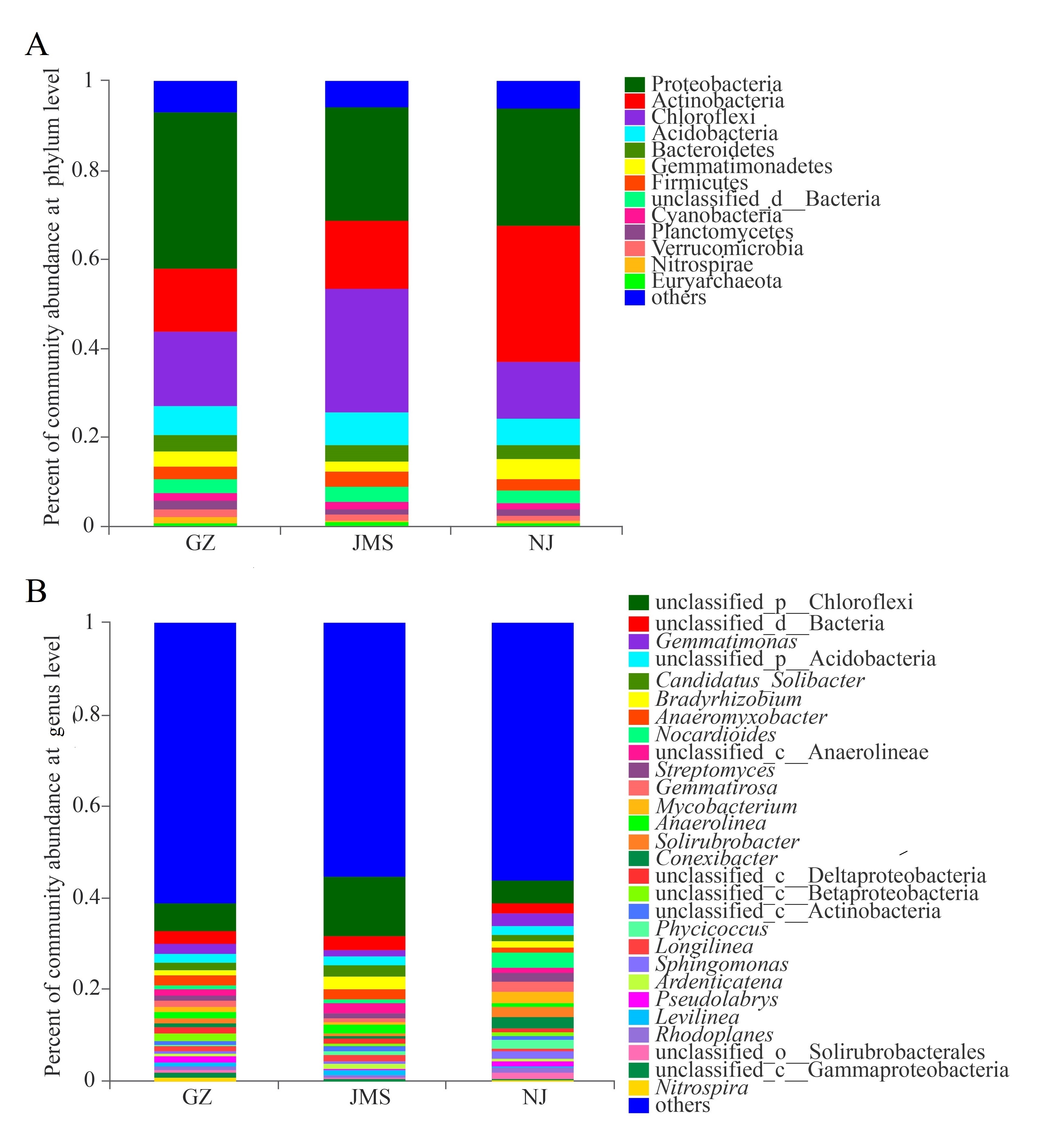


Fig. S1. Relative abundance of main microbial phyla (A) and genera (B) in soils of three rice cropping agroecosystems. GZ: rice double cropping in Guangzhou; JMS: rice single cropping in Jiamusi; NJ: rice–wheat rotation in Nanjing.


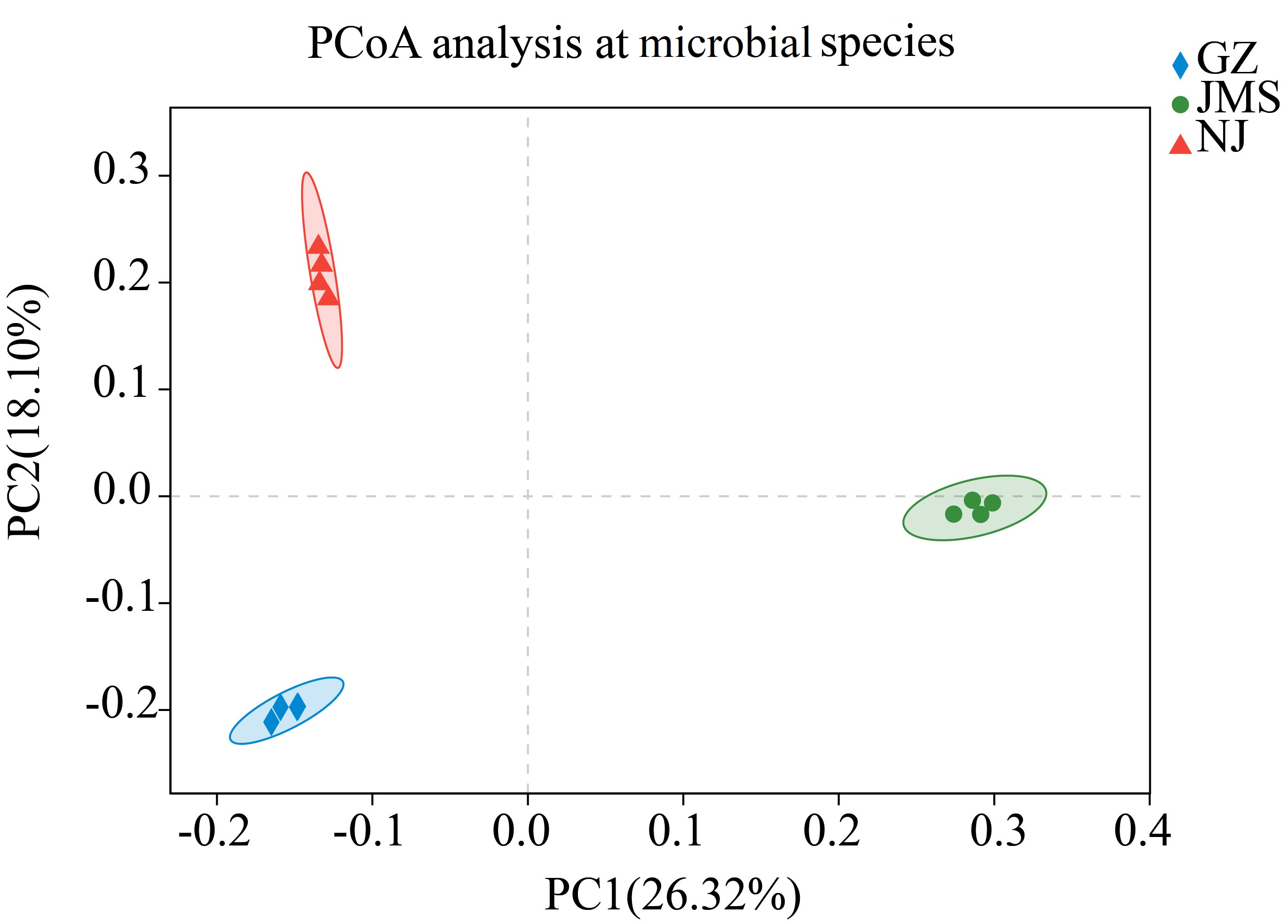


Fig. S2. Principal coordinate analysis (PCoA) of soil microbial community based on Bray–Curtis distance matrices. The percentage represents the explanatory value of the PCoA axes to the difference in sample composition; distances between symbols on the ordination plot reflect relative dissimilarities. GZ: rice double cropping in Guangzhou; JMS: rice single cropping in Jiamusi; NJ: rice–wheat rotation cropping in Nanjing.


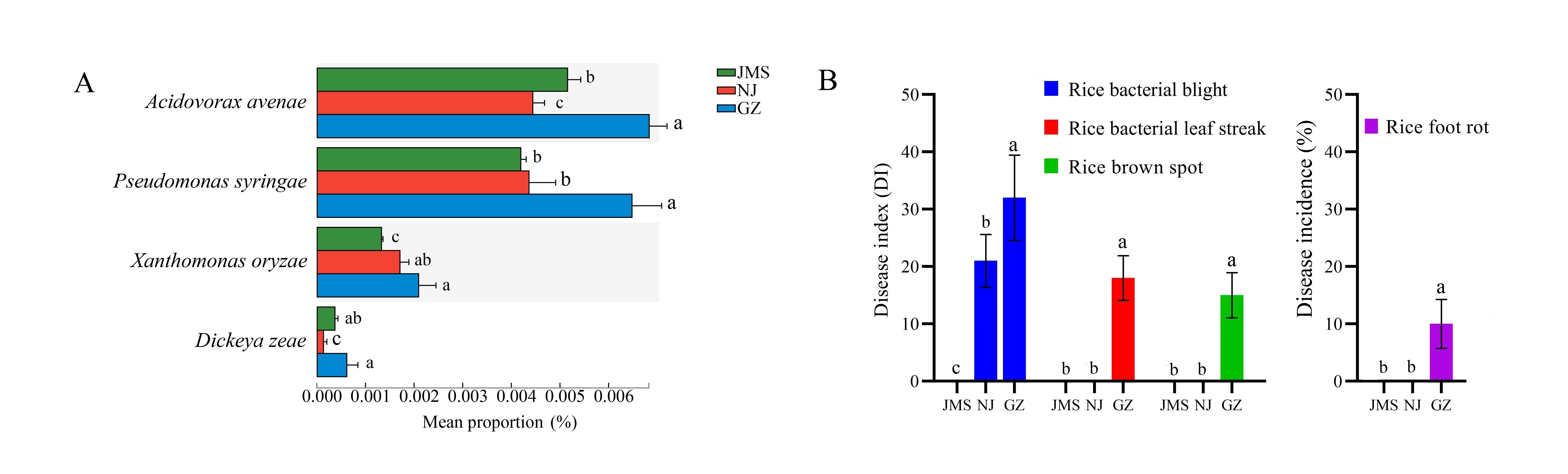

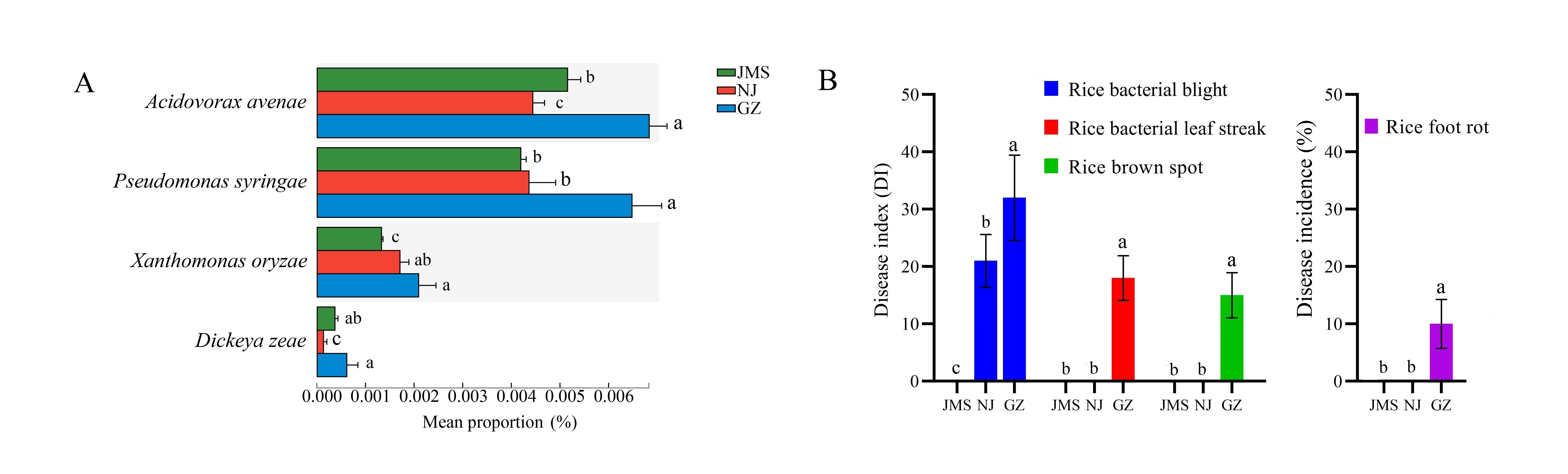


A

B

Figure S3. Relative abundance of potentially pathogenic bacteria (A) and disease index for four rice diseases in the three agroecosystems in China. Different letters above the bars indicate a significant difference (*p* < 0.05) according to Kruskal-Wallis H test. GZ: rice double cropping in Guangzhou; JMS: rice single cropping in Jiamusi; NJ: rice–wheat rotation cropping in Nanjing..


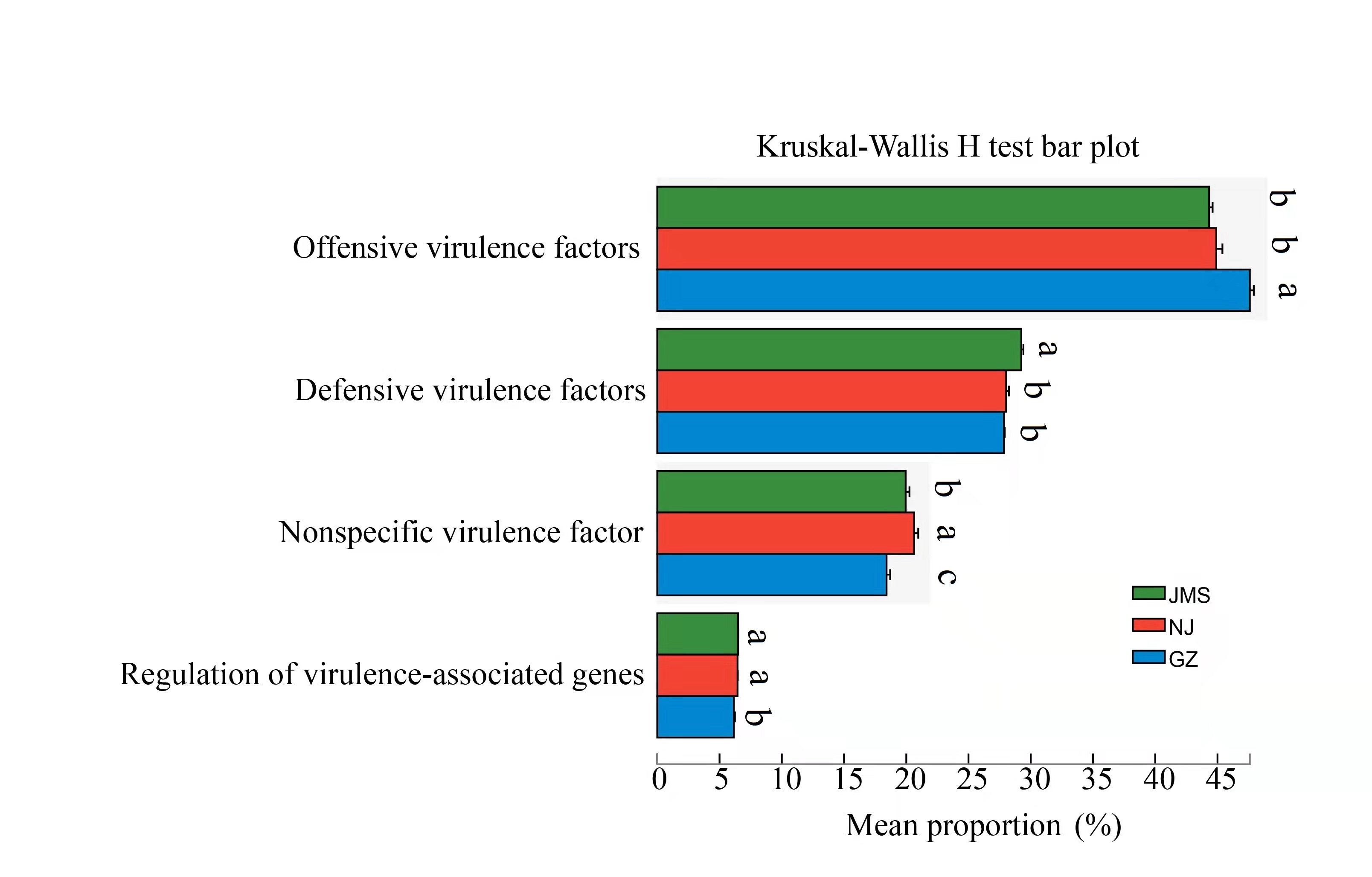


Fig. S4. Relative abundance of genes related to xenobiotics biodegradation and metabolism in viruses and bacteria in soils of three rice agroecosystems. GZ: rice double cropping in Guangzhou; JMS: rice single cropping in Jiamusi; NJ: rice–wheat rotation in Nanjing. Different letters above the bars indicate a significant difference (*p* < 0.05) according to Kruskal-Wallis H test.


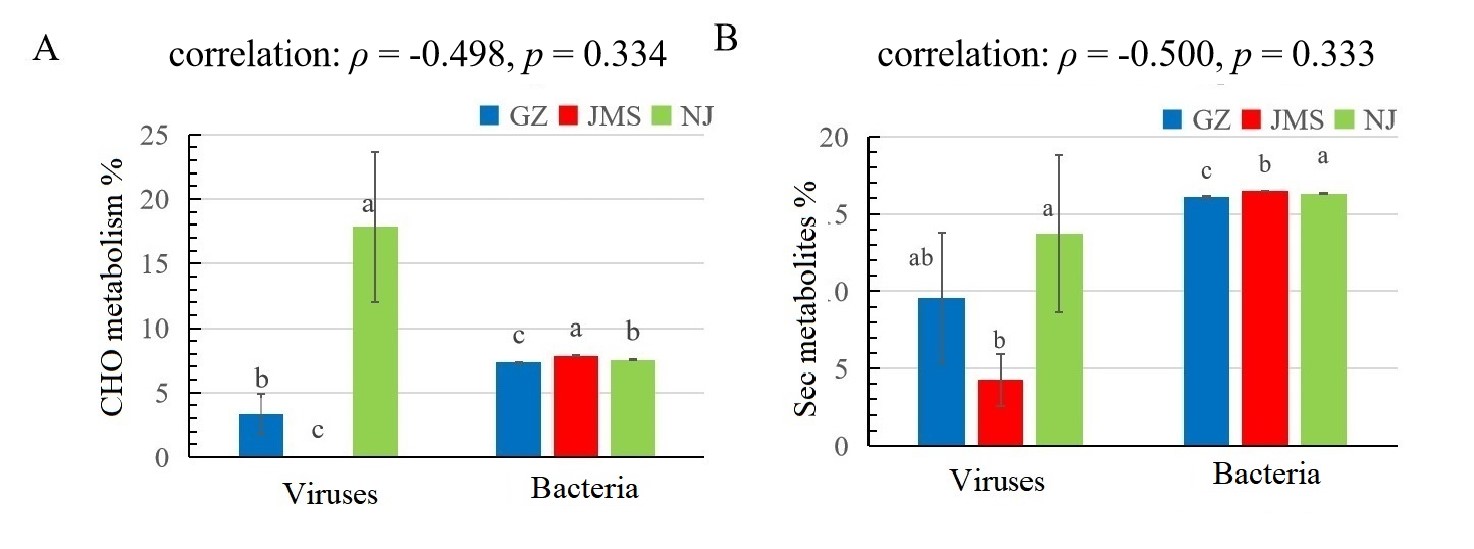


Fig. S5. Relative abundance of genes related to carbohydrate (CHO) metabolism (A) and biosynthesis of secondary (Sec) metabolites (B) in viruses and bacteria in soils of three rice agroecosystems. GZ: rice double cropping in Guangzhou; JMS: rice single cropping in Jiamusi; NJ: rice–wheat rotation in Nanjing. Different letters above the bars indicate a significant difference (*p* < 0.05) according to Kruskal-Wallis H test.


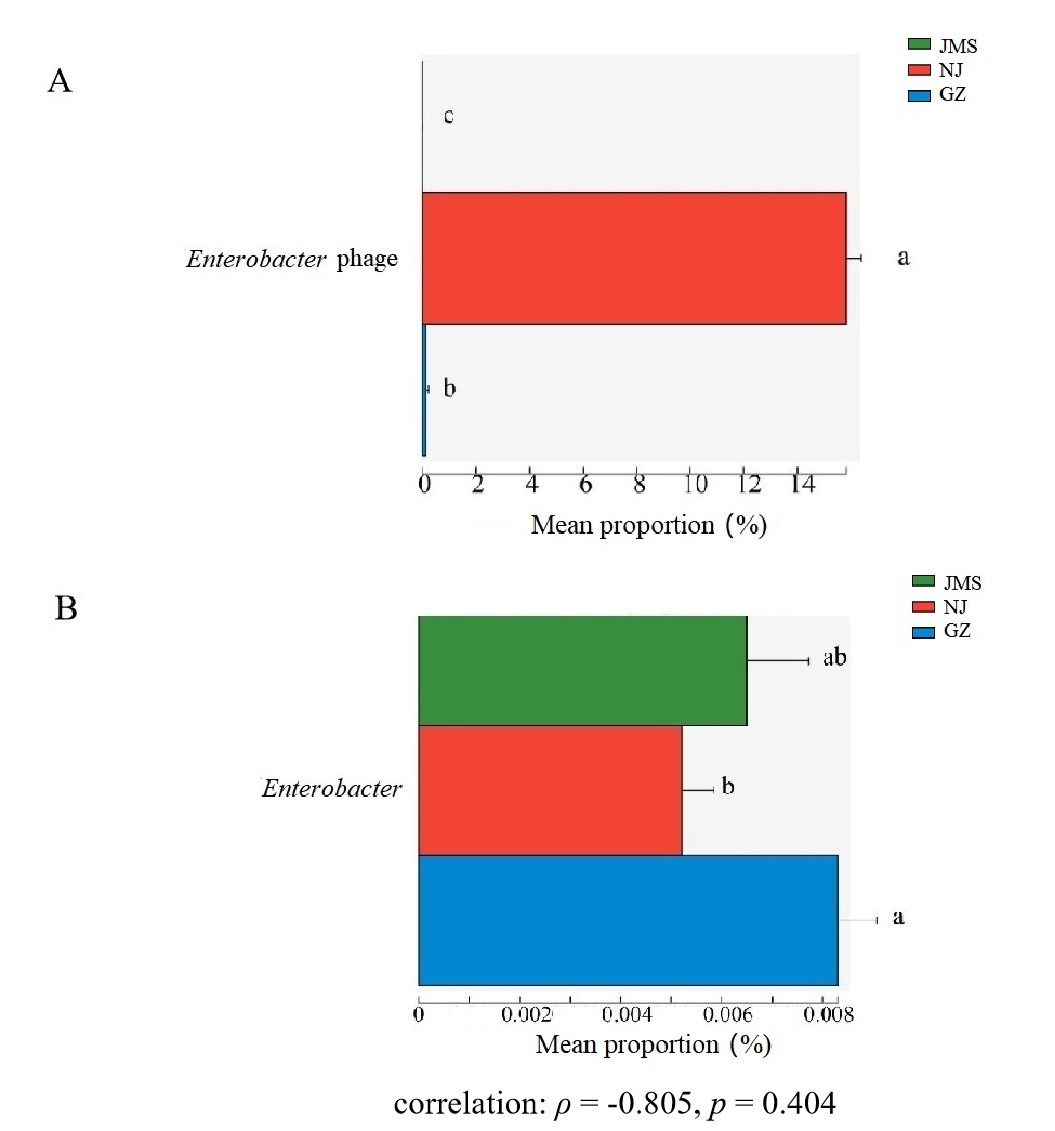


Fig. S6. Relative abundance of *Enterboacter* phage among soil viruses (A) and relative abundance of *Enterboacter* among soil bacteria (B) in the three rice agroecosystems. GZ: rice double cropping in Guangzhou; JMS: rice single cropping in Jiamusi; NJ: rice–wheat rotation in Nanjing. Different letters above the bars indicate a significant difference (*p* < 0.05) according to Kruskal-Wallis H test.


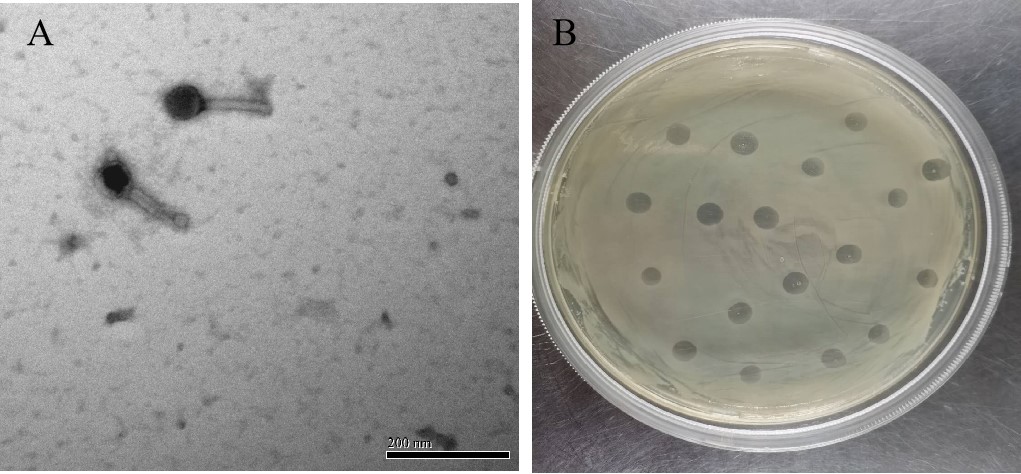


Fig. S7. (A) Transmission electron micrograph of *Enterobacter* phage-NJ. (B) Plate lytic experiment of *Enterobacter* phage-NJ against nitrogen-fixing *Enterobacter cloacae.*
